# Vertical Transmission of Hepatitis B and C—Then and Now—A Comprehensive Literature Systematic Review

**DOI:** 10.3390/v17101395

**Published:** 2025-10-20

**Authors:** Ruxandra Dobritoiu, Daniela Pacurar, Raluca Maria Vlad, Doina Anca Plesca

**Affiliations:** 1 “Carol Davila” Department of Pediatrics, University of Medicine and Pharmacy, 020021 Bucharest, Romania; daniela.pacurar@umfcd.ro (D.P.); raluca.vlad@umfcd.ro (R.M.V.); doina.plesca@umfcd.ro (D.A.P.); 2 “Grigore Alexandrescu” Emergency Children’s Hospital, 011743 Bucharest, Romania; 3 “Matei Bals” National Institute of Infectious Diseases, 021105 Bucharest, Romania

**Keywords:** hepatitis B, hepatitis C, vertical transmission, pregnancy, newborn, antiviral therapy

## Abstract

Background: According to a WHO global hepatitis report, the global prevalence of hepatitis B in 2022 was 254 million and for hepatitis C it was 50 million. The estimated number of people newly infected by viral hepatitis declined from 3 million in 2019 to 2.2 million in 2022. Of these, 1.2 million are hepatitis B infections and nearly 1.0 million are hepatitis C infections. Regarding vertical transmission, it is estimated that 4 to 5 million children are infected worldwide every year from HBV-positive mothers. The United States declared that hepatitis C is the commonest chronic blood-borne infection, with an increase in HCV birth infections from 1.8 to 4.7 per 1000 births. Objectives: This systematic review focuses on highlighting the most suitable screening methods and maternal interventions to prevent HBV/HCV mother-to-child transmission, as well as the appropriate prophylactic strategies for newborns. Materials and methods: We searched a medical database (PubMed) to find papers regarding mother-to-child transmission of hepatitis B and C. Inclusion criteria were human-based studies, studies with large cohorts of subjects, studies conducted in different parts of the globe and position papers from various international associations. Exclusion criteria were non-human-based studies and non-English publications. To present and synthesize results we made use of thematic analysis and narrative synthesis. Results: We included 103 publications. For hepatitis B, the combination of maternal antiviral therapy during pregnancy and timely administration of HBV vaccine alongside HBIG to the newborn has proven to be highly effective in lowering transmission rates. Hepatitis C vertical transmission lacks an effective vaccine or immuno-prophylaxis, turning prevention strategies into a continuous battle. Conclusions: Vertical transmission of hepatitis B and C continues to be a major contributor to the global burden of chronic viral hepatitis. Strengthening prenatal care programs, improving access to diagnostic and therapeutic resources and enhancing public health policies are essential to curb vertical transmission of both hepatitis B and C.

## 1. Introduction

Viral hepatitis continues to represent an enormous burden to both patients and health systems worldwide, turning the need for prevention and prophylaxis methods into an urgent public health issue. Despite advances in screening and prevention measures, mother-to-child transmission remains a critical pathway for new infections. Strengthening prenatal care programs, improving access to diagnostic and therapeutic resources and enhancing public health policies are essential. This review focuses on mother-to-child transmission of hepatitis B and C, with emphasis on seroprevalence, risk factors during pregnancy and birth, prophylaxis methods and recommendations for further care.

Overall, about 304 million people were living with viral hepatitis B or C in 2022, so one of the key objectives of the WHO is to eradicate viral hepatitis by 2030 [1,2]. These two viruses are responsible for 96% of all deaths from hepatitis [3,4]. The World Health Organization (WHO) estimated that there are 1.5 million new infections each year with HBV [5,6], particularly prevalent in East Asia and South Africa where it affects approximately 10% of the adult population [5]. In Europe, the estimated prevalence in 2020 was low for western areas (1%) and less than 5% for eastern regions, but these numbers might have changed because of massive migrations [4]. For HCV, the prevalence is estimated at 2.3% in Europe, the most affected area being the Eastern Mediterranean [4], whereas in the United States, 3.5 million people suffer from chronic hepatitis C [7,8].

Regarding vertical transmission, it is estimated that 4 to 5 million children are infected worldwide every year from HVB positive mothers [9]. The United States declared that hepatitis C is the commonest chronic blood-borne infection [8,10] with results from a nationwide study from 2009 to 2017 which reported an increase in HCV birth infections from 1.8 to 4.7 per 1000 births [7]. A study involving 1.7 million pregnant women from Florida concluded that HVB prevalence was higher in Asian Americans and African Americans compared to Caucasians [10]. The prevalence of HCV-positive pregnant women in Spain is estimated at 1.4%, but the rate of vertical transmission is quite low (1–8%) [4].

In June 2016, a collaborative effort by the United States Centers for Disease Control and Prevention, the WHO and the ZeShan Foundation led to the first International Roundtable Summit on Funding for Elimination of Viral Hepatitis. The main goal was to develop easy-to-use diagnostic tools and facilitate diagnosis in health facilities with limited laboratory infrastructure, all performed at a cost-effective price, using economic analysis regarding treatment and care of patients with chronic hepatitis from many areas. For example, in Egypt, the prevalence of hepatitis C is very high and health care costs consume 4% of the total health expenditure. Last year, in northwestern Ethiopia, overall prevalence of HBsAg was 6% and that of anti-HCV antibodies was 2.4% [11]. In China, back in 2017 when costs of hepatitis B treatment were not sustained by health care insurance, tenofovir therapy cost USD 2929 per year (almost 5 times more than HIV treatment) [8]. So, national immunization programs, easy access to health care facilities especially for those at high risk, lowering the prices for medicines and developing government programs which partially or fully sustain therapy cost are the main keys in fighting the battle against hepatitis.

**Figure 1:**
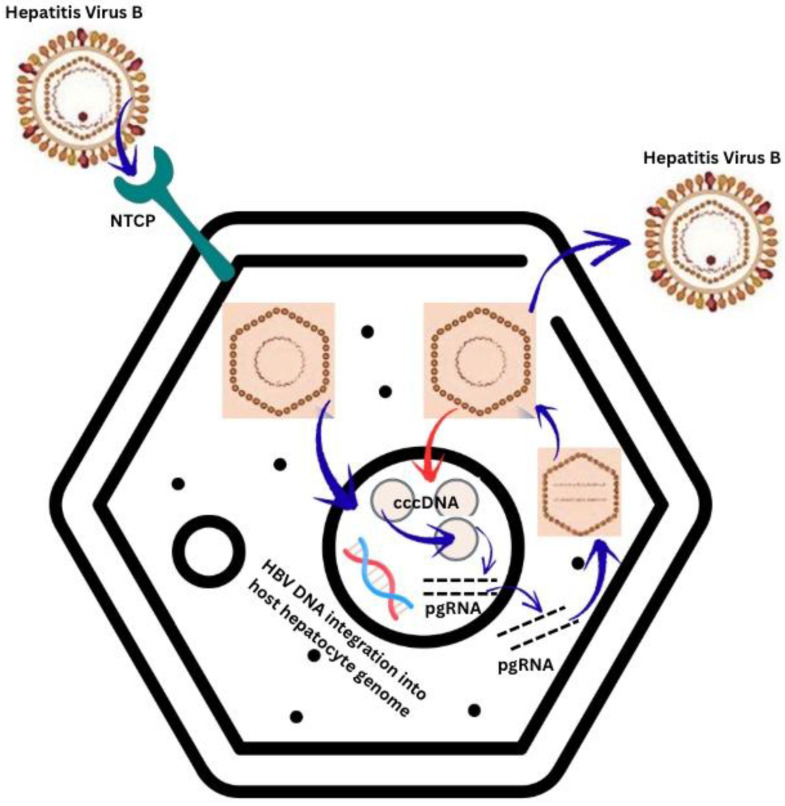
Hepatitis B Virus—Mechanism of replication [3,12,15,17]. NTCP—sodium taurocholate co-transporting polypeptide. pgRNA—pregenomic ribonucleic acid.

HBV is an enveloped virus from the Hepadnaviridae family and consists of a double-stranded circular DNA genome [3,12,13,14]. The replication cycle of HBV is briefly exhibited in Figure 1.

Clinical features of HBV infection range from asymptomatic to acute fulminant hepatitis, with an incubation period ranging from 40 to 90 days [13,15,16].

Transmission of HBV is through cutaneous or mucosal exposure to infected blood or body fluids [3,13,15,18]. The hepatitis B core antigen (HBcAg) comes from the nucleocapsid core, which is surrounded by a lipoprotein coat produced and released in massive amounts in the blood stream—the hepatitis B surface antigen (HBsAg) [3,12,14,15,18,19]. Viral replication occurs primarily in the liver and an infectivity marker of HBV infection is HBeAg, a viral protein produced by the HBcAg reading frame [3,12,15,18]. HBV will integrate and release its own DNA into the genome of host hepatocytes, where it establishes a closed circular pool, named covalently closed circular DNA (cccDNA), responsible for infection persistence [12,13,14].

There are five phases regarding the natural course of HVB infection: immune tolerance, immunoreactivity, inactive carrier, HBeAg-negative phase and HBsAg-negative phase [9,20]. The first phase occurs during perinatal infection and is marked by very high levels of viral DNA load, positive HBsAg and HBeAg. The second phase occurs in children infected at birth or early childhood, debuts after two to three decades of life and is marked by moderate viral load, high ALT and positive HBsAg. The third phase, which goes on for decades, is known for low viral load and ALT values. The fourth phase is characterized by progression of liver fibrosis, with high risk of developing hepatocellular carcinoma (HHC). The last infection phase, known as “occult” chronic B hepatitis, is marked by absence of HBsAg and normal ALT levels, but immunosuppression status, such as in pregnancy, may cause reactivation of infection [9,14,20,21,22].

The highest risk of vertical transmission seems to be in the immune tolerance phase, probably because of the severely elevated viral load (10^9^ UI/mL). The lowest risk for mother-to-child transmission is present in the fifth phase because of unidentifiable viral load and negative HBsAg [9,20,23,24]. The serological and molecular markers of HBV infection are briefly exhibited in Table 1.

HCV, a small, encapsulated, single-strand RNA virus, is part of the Flaviviridae family and is mostly transmitted via percutaneous inoculation [10,25,26,27]. Other ways to contract HCV are through sexual intercourse, household behaviors, occupational risks and mother-to-child transmission [3,4,26,27].

**Figure 2:**
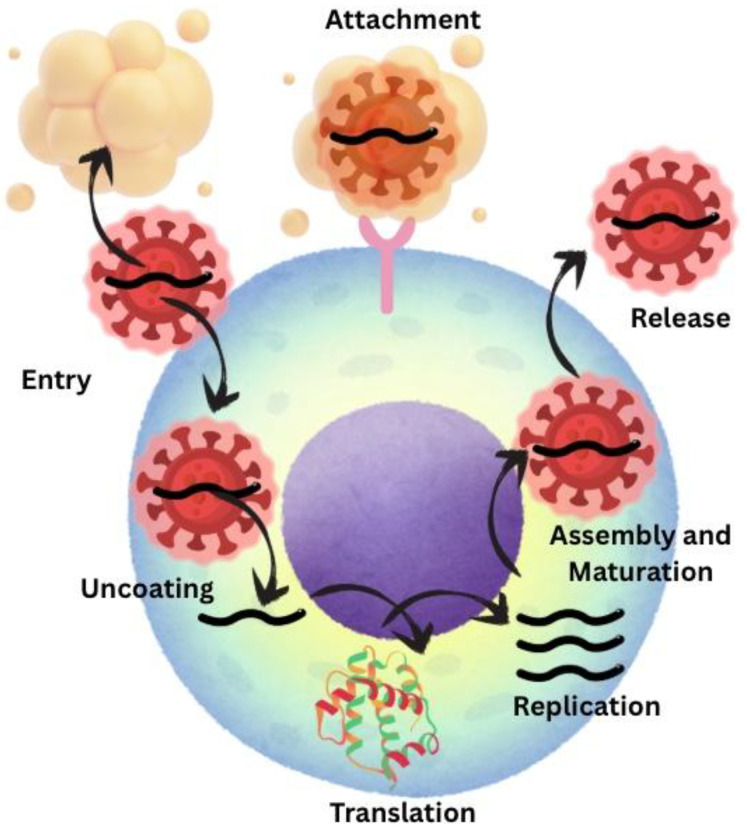
Replication mechanism for HCV [28].

Replication of HCV includes seven steps: 1—attachment of viral particle to the cell; 2—endocytosis into the cell by ditching the lipid external coat; 3—fusion of cellular and viral membranes which releases the genomic RNA into the cytoplasm; 4—translation of genome RNA into proteins; 5—replication of HCV RNA genome; 6—assembly and maturation of virions; 7—release of mature virions from the cell (Figure 2) [28].

Compared to HBV, vertical transmission of HCV is lower (3–14%) [25,26,29] and there is a significant difference between pregnant women positive for anti-HCV antibodies and those positive for HCV-RNA (1.7% compared to 4.3%) [26,29]. Incidence of acute HCV infection during pregnancy and development of fulminant hepatitis is low [3]. Development of antibodies against HCV does not produce immunity against this disease [3,7,27,30].

Nowadays, the leading source of infection in pediatric patients from developed countries is vertical transmission, with prevalence of HCV infection among pregnant women resembling that of the general population [25,26]. HCV is considered the main cause of chronic viral hepatitis among children [26].

## 2. Materials and Methods

Published literature abounds in information about HBV and HCV vertical transmission, as well as in data regarding prophylaxis measures.

This literature review is based on PubMed papers published in the last 23 years (2002–2025) and concerns characteristics of mother-to-child viral transmission during pregnancy and after delivery, with a primary interest in prevention methods. The following particular search terms in different combinations with filters for case reports, clinical trials and reviewed articles were used in order to find the most suitable papers for this review: “hepatitis B”, “hepatitis C”, “vertical transmission”, “mother-to-child transmission”, “prophylaxis”, “antiviral therapy”, “vaccination”.

The review process followed the Preferred Reporting Items for Systematic Reviews and Meta-Analyses (PRISMA) guidelines. The selection of studies is summarized in the PRISMA flow diagram (attached in the Supplementary Material).

Data was also sought by a list of variables, including type of study and details about demographics. We first screened titles and abstracts using predefined criteria, then assessed the full text of potentially eligible studies. To collect data from these papers, we analyzed the entire content, through qualitative and quantitative assessment, so we could interpret information in our narrative report. To decide which results to collect, we clearly identified the necessary information through extensive and careful reading of each article, then extracted it from all papers included.

Inclusion criteria were human-based studies, studies with large cohorts of subjects, studies conducted in different parts of the globe and position papers from various international associations. Exclusion criteria were non-human based studies and non-English publications.

To present and synthesize results we made use of thematic analysis and narrative synthesis. We searched for and selected papers and studies that were relevant for HBV/HCV MTCT and we extracted suitable information from each included paper. We looked for similar concepts across all papers, extracted suitable data and combined the findings, which we then interpreted to draw our own conclusions.

Based on the keyword search, we encountered 163 publications. We excluded 60 of the articles, either because the inclusion criteria were not met, because duplicates were found or because they were not written in English.

## 3. Screening Techniques

### 3.1. Screening of Pregnant Women with HBV Infection

All pregnant women should benefit from HBV infection testing in their first trimester in each pregnancy, regardless of their vaccination status [20,29,31,32,33]. In China, back in 2011, a national integrated prevention program of mother-to-child transmission (MTCT) of HIV, HBV and syphilis was implemented and provided an essential tool for further management of these infections [29]. In Singapore, antenatal screening consists in detection of HBeAg and anti-HIV antibodies [31].

The Centers for Disease Control and Prevention has elaborated a series of strong recommendations for the prenatal identification and management of HBV-infected pregnant women [15]. Timing of prenatal HBsAg screening is also crucial because it has a massive impact on MTCT, as infants born to mothers with high viremia load (HBV-DNA > 10^6^ UI/mL) or positive HBeAg have a 10–15% higher risk of developing HBV infection, despite receiving postexposure prophylaxis [21,24,32,34,35]. A study from University Hasanuddin, Indonesia including a total of 943 pregnant women concluded that quantitative serum HBsAg levels may be used as a predictor of viral HBV load in HBeAg-positive subjects, meaning that HBV-DNA levels are not necessary to measure if levels of HBsAg are low [19].

All HBsAg-positive mothers should be tested for HBeAg, anti-HBe antibodies, HBV-DNA levels and biochemical markers of liver function immediately, as maternal antiviral therapy is an effective recommendation for preventing MTCT [15,29,33,36,37,38]. Presence of active hepatitis or liver fibrosis puts everything in a different light—specific recommendations are made for this group of pregnant HBV-positive women (Table 2).

Newborns of HBV-positive mothers must be tested at 2 months after the final HBV vaccine dose was administered, meaning at approximately 12 months of corrected age. Screening evaluates HBsAg and anti-HBs antibodies. No testing of anti-core antibodies should be performed in infants, because they can passively pass through the placenta and be detected until 24 months of age in a child’s circulation. Depending on the child’s HBs antibody load, a fourth additional vaccine dose can be given in order to increase protection against HBV infection [21,24,32,39,40].

### 3.2. Screening of Pregnant Women with HCV Infection

According to the American Association for the Study of Liver Diseases (AASLD), all pregnant women should be tested for HCV infection at the time of first prenatal visit, because the risk of MTCT increases and severe potential complications regarding delivery and fetal outcomes may occur when maternal viral load is higher than 10^5^ copies/mL [41,42,43,44]. Although most infected women do not develop HCV-related clinical manifestations during pregnancy, intrahepatic cholestasis was described to be 20 times more frequent in HCV-positive pregnant women [25,42,43,44,45]. Infants of HCV-positive mothers with high viremia load are more likely to develop delayed fetal development, smaller head circumference [41], be born prematurely (60% higher risk) and require ventilatory support and admission to a neonatal intensive care unit (NICU) [25,42,45,46]. There is also evidence of increased risk of gestational diabetes mellitus [42] and pregnancy anemia [41] in high-level HCV-RNA pregnant women. The rate of HCV MTCT increases if there is HIV co-infection [25,26,42,43,44,45].

## 4. Mother-to-Child Transmission of Viral Infection

### 4.1. Vertical Transmission of HBV Infection

Mother-to-child HBV transmission was first noted in 1862 by Saint-Vel in a pregnant woman from Martinique. But it was not until the late 1950s that vertical transmission was documented with the discovery of infants developing hepatitis during the first months of life. Later, a prospective study conducted in the United States and Europe concluded that 66% of infants from mothers with acute HBV infection developed viral hepatitis within six months of life [20]. Vertical transmission of HBV can occur in any stage of pregnancy, from in utero to breastfeeding stages [20,21,23,32,47,48]. In an update paper regarding MTCT of HVB, Fillipo Villa et al. proposed a useful schematic algorithm of pregnancy-related HBV infection, dividing the potential timeframe of transmission into three periods: during gestation (2nd to 32nd week), during delivery (32nd to 40th week) and after delivery, each one consisting in different pathways of mother-to-child viral transmission [20].

#### 4.1.1. Transplacental Transmission of HBV Infection

**Figure 3:**
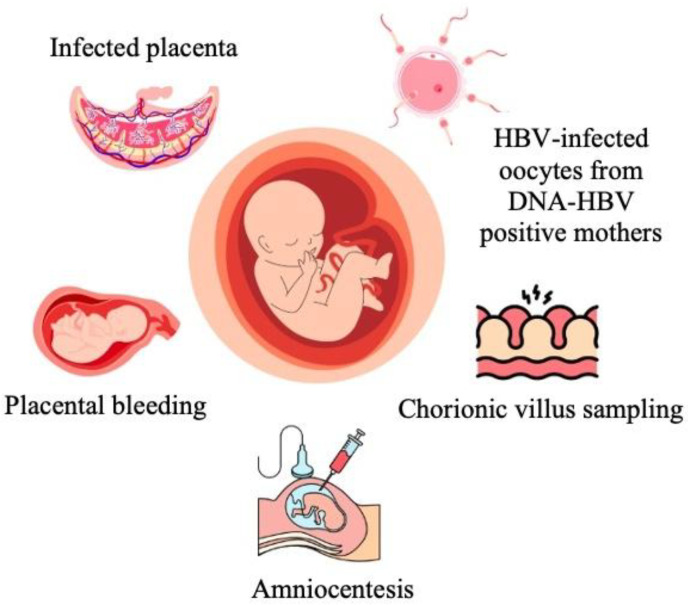
Pathways of in utero HBV vertical transmission [20,21,23,32].

In utero infection, which could explain immuno-prophylaxis failure, occurs through placental cells, transplacental leakage of maternal blood, during amniocentesis, through infected maternal peripheral blood monocellular cells or through germline cells [20,21,23,32,48,49,50] (Figure 3). It is well known that HBV and HBsAg cannot traverse the placenta, but HBeAg, being smaller, is able to do so [9,32].

Transplacental leakage is the commonest way of IUT and can occur early in pregnancy due to immature placenta or later because of uterine contractions [20,32].

**Figure 4:**
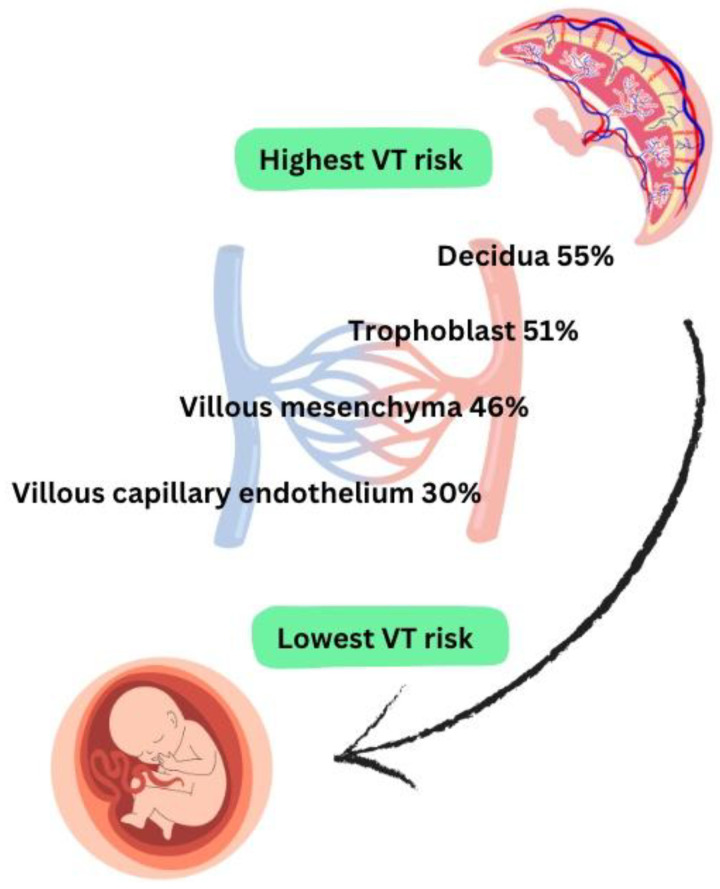
HBV infection of placental cells [32,52].

The placenta can also become infected—in a study by Chen et al. which examined HBV infection in 157 placental tissues, it was established that the infection rate was higher in decidua cells and in trophoblastic cells (55% and 51%) compared to villous mesenchymal cells and villous capillary endothelial cells (46% and 30%) [20,32]. This study is supported by another one, Xu et al. [51], which reported similar results—the HBV infection rate gradually decreases from the maternal to fetal placental layer, therefore HBV can cross the entire placental barrier and can replicate in all types of placental cells, but infection of villous capillary cells is associated with the highest VT risk [20,32] (Figure 4).

Invasive obstetrical procedures during pregnancy, such as amniocentesis, are associated with a higher risk of HBV VT [20,21,24,32]. Amniocentesis exposes the fetus to HBV in two different ways: either the fetus swallows contaminated amniotic fluid or a maternal–fetal blood exchange takes place [4,5,20]. Chorionic villus sampling, placing fetal scalp electrodes, vigorous suctioning of newborn airways at birth or any other procedure that breaks skin and mucosal barriers should be avoided [20,24]. In a case–control study, prevalence of MTCT of HBV was significantly higher in pregnant women with elevated viral load [21]. A Chinese study enrolling 642 infants born to HBsAg-positive mothers concluded that the risk of VT was higher in those who underwent amniocentesis and were born from mothers with viral load of more than 2 × 10^6^ UI/mL (*p* = 0.006) [24].

Another pathway of IUT is via infected maternal peripheral blood monocellular cells (PBMCs) because infected cells serve as carriers of HBV from mother to fetus [20,32,49,51]. A recent case–control study which evaluated HBV serology in infants from 312 HBsAg-positive mothers demonstrated that mother-to-child PBMC transfer represents an important risk factor for HBV VT [20].

A fifth way of IUT of HVB is through germline cells, via oocyte or embryos from HBsAg-positive women during in vitro fertilization [20,23,32,53,54,55]. Jin L et al. reported two mothers with HBsAg-positive oocytes who gave birth to infected infants [54]. HBV-DNA was found in embryos from couples with at least one HBV-infected member [20,53,54]. In a case–control study by Xiaoling Hu et al. from 2024, which included 167 couples with HBV-positive oocytes or embryos and 91 couples with HBV-negative oocytes or embryos, it was determined that HBV-DNA oocytes or embryos may not result in VT of HBV to offspring of HBV carriers [53]. This study is supported by another one from Jin et al., which reported that infants born from in vitro fertilization with HBV-positive oocytes or embryos were not infected with HBV [20,54]. The fertilization method also has a significant impact on the viability of HBV in infected oocytes [20,53,54].

Regarding sperm samples from HBV-DNA-positive males, a study conducted by Cai et al. described eight fathers with HBV viral load who had non-HBV-infected offspring via in vitro fertilization; in this study, infected infants resulted from HBV-positive mothers, regardless of the father’s viral status [32]. Another study supports the above statement—Qun Xi et al. obtained sperm samples from three HBV-DNA-positive males; none of their infants was infected with HBV (but the mothers were HBsAg-negative) [20].

#### 4.1.2. Perinatal Transmission

Mother-to-child HBV transmission during childbirth can occur in three ways: instrumental trauma during delivery, maternal–fetal blood micro-transfusion and neonatal contact with maternal vaginal secretions or vaginal cells [20,32,35]. Perinatal MTCT is the most common route of HBV vertical transmission, reaching up to 40% of cases [20,21]. High maternal HBV load alongside positive HBeAg is considered to significantly increase the transmission rate in the perinatal stage [21,37]. Positive HBsAg and high levels of viral HBV load were found in the umbilical cord, amniotic fluid and vaginal fluid (96%) but also in the gastric content (90%) and serum (lasting 2–3 months) of neonates from infected mothers [21,35,36,37,56]. A study of 447 infants from HBsAg-positive and HBeAg-negative mothers found higher rates of HBV VT for those delivered vaginally than in newborns delivered by c-section (25% in comparison with less than 10%) [20].

#### 4.1.3. Postnatal Transmission

Mother-to-child HBV transmission after birth can occur in two ways: through breastfeeding or contact with maternal saliva [20,21,23,24,32,57]. Although possible, after birth transmission is less common (<7%) [20] because the viral load in breast milk and maternal saliva is very low compared to the blood [21]. Regarding breastfeeding, there are three major concerns: Is breastfeeding a risk factor for postnatal HBV transmission? Does breastfeeding interfere with the immune response after vaccination? Can we recommend breastfeeding for a mother under antiviral therapy [24,57]?

Lesions in the breast tissue with serous exudate or cracked nipples could be a source of HBV MTCT, more so because of the fragile nature of the neonate’s gastrointestinal mucosa [20,23]. But, keeping in mind that the gastrointestinal tract is not the primary route of HBV transmission and that there is no relevant evidence-based data that breastfeeding should be considered a risk factor for MTCT of HBV [20,21,23,24,32], nowadays guidelines, including those of the WHO, recommend that all infants from HBV-positive mothers should be breastfed for 4 to 6 months [20,23,24,57]. Benefits of breastfeeding exceed any potential risk of infection [23,24,57]. In one study comparing breastfed infants versus formula-fed infants, there was evidence of the protective role of the mother’s milk against chronic HBV infection through maternal antibodies present in breast milk [20,32].

Regarding interference of breastfeeding with immune response after HBV vaccination, Wang et al. elaborated a study including 230 infants, who received immuno-prophylaxis at birth. Subjects were followed up for one year and there was no significant difference in incidence of immuno-prophylaxis failure between breastfed and formula-fed babies [24].

The main concern about breastfeeding mothers on antiviral therapy is the child’s long-term exposure to these drugs, which may have an impact on growth and development [24,58,59]. However, AASLD currently states that HBV-positive mothers on tenofovir therapy can and should breastfed because only small amounts of the drug have been found in breast milk of women undergoing antiretroviral therapy [24].

### 4.2. Vertical Transmission of HCV Infection

**Figure 5:**
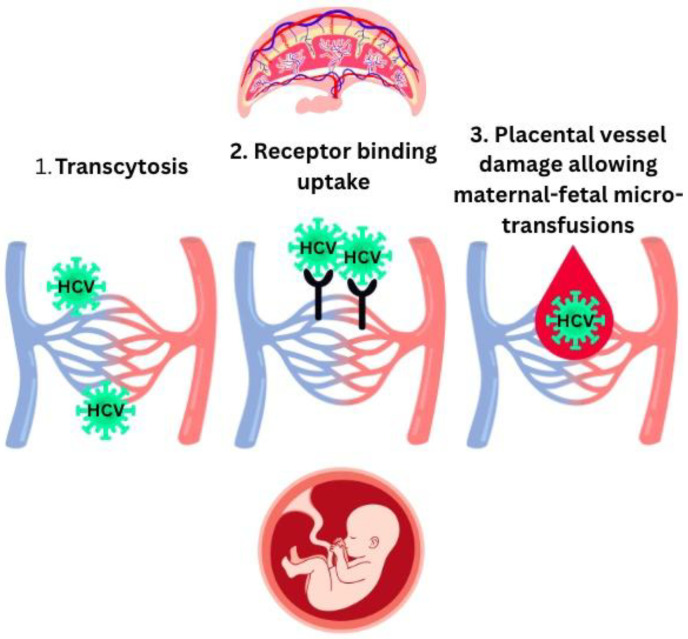
Pathways of HCV IUT transmission [27,32,60].

The leading cause of pediatric HCV infection is MTCT during pregnancy or delivery. It is estimated that 3% to 10% of HCV-positive pregnant women transmit the infection to their infants [10]. Intrauterine transmission of HVC seems to be lower (30%) than perinatal transmission (40–50%) [32]. Approximately 1/3 of IUT HCV transmissions occur between 25 and 36 weeks of gestation [7,46,48]. According to a meta-analysis, VT of HCV is estimated at 5.8% and the risk doubles if there is HIV co-infection [46]. The fetus may be exposed to HCV through two pathways: either free virions and other virus-related cells directly cross the placenta or because large amounts of HCV copies infect the placenta during pregnancy, so a severe inflammatory response leads to placental barrier damage and creates a breach (Figure 4) [7,30,32,44,46]. Le Campion et al. proposed that HCV MTCT occurs by transcytosis [27,32], whereas Giugliano et al. discovered that the primary trophoblast, which is a specialized epithelial layer of the placenta, expresses receptors for HCV [32,60] (Figure 5).

During childbirth, transmission of HCV can occur through various pathways, all involving exposure of the neonate to maternal infected blood [7,44]. There is no evidence that c-section birth prevents MTCT in HCV-positive HIV-negative pregnant women [7,10,25,32,43,46], therefore there are no guideline recommendations for these cases.

Regarding breastfeeding for HCV-positive women, the question remains the same as in the case of HBV—do breastfed infants have a higher risk of infection? Small amounts of HCV-RNA can be detected in breast milk, but there is no evidence to consider breastfeeding a risk factor for maternal transmission of HCV. In the case of breaches, as infected maternal blood could come into contact with the infant (like cracked nipples), there is a concern for HCV MTCT [10,25,32]. In a study conducted by Belopolskaya et al. from 2021, none of the 76 breast milk samples from HCV-positive mothers contained HCV-RNA and more than 50% of them had high HCV load [9]. Another study conducted by Tovo et al. suggested that human milk is able to inactivate HCV because lipase breaks down fats from the viral envelope [25,32].

## 5. Risk Factors for Mother-to-Child Viral Transmission

### 5.1. Risk Factors for HBV Vertical Transmission

**Figure 6:**
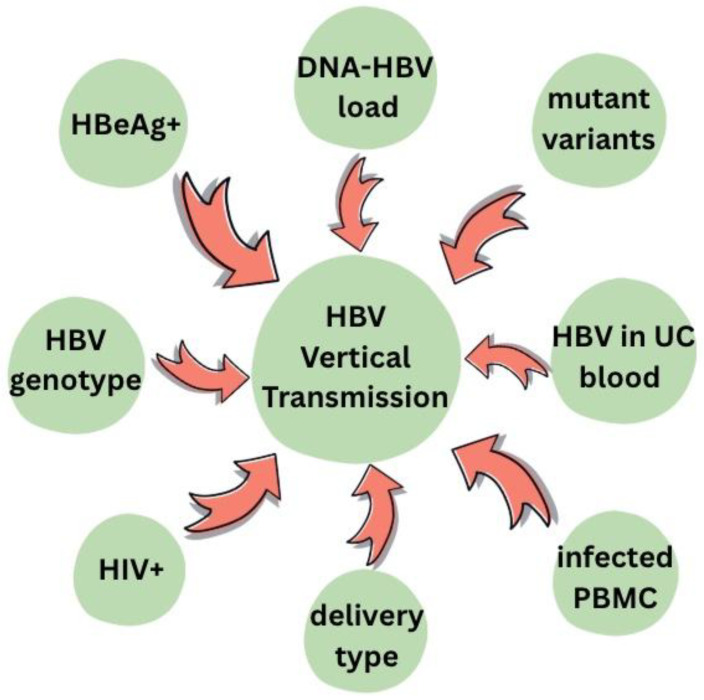
Risk factors for mother-to-child HBV transmission [20,61,62].

Various factors are identified (Figure 6) to increase the risk of HBV MTCT, among them being positive status for HBeAg, co-infection with HIV or type of delivery [20,61,62].

#### 5.1.1. Positive Status of HBeAg

HBeAg is a secretory protein with immunomodulatory functions that is not required for HBV replication, but it is a serological marker of elevated viral replication, with an essential role in chronic HBV hepatitis [20,31,61,63]. It is associated with high risk of vertical transmission and is responsible for establishment of chronic HBV infection after MTCT [20,31,61,64].

Fillip Villa et al. mentioned a case–control study carried out in India in 2011 and published in *Indian J. Gastroenterol.*, in which vertical transmission was encountered in 65% of newborns from HBeAg-positive and HBV-DNA-positive mothers [20]. Furthermore, HBeAg is the only viral-related marker that can cross the placenta (because it is only 17 kDa), so it can generate infant immunotolerance to HBV, which leads to premature chronic infection [20,32,64].

Kuen-Nan Tsai et al., in a paper published in 2021, regarding HBeAg and viral persistence, supported that HBeAg+ carrier mothers were much more likely to transmit the virus to their offspring. The article mentions that infants born to HBeAg-negative and HBV-positive mutant mothers usually develop self-limited acute infections and do not become chronic HBV carriers. So, even in the presence of mutant viral variants, chronic infection is still linked to the positive status of HBeAg [13,61,63].

#### 5.1.2. HBV-DNA Viral Load in Maternal Blood

High maternal viral load is considered an independent risk factor and an important predictor of HBV MTCT, therefore having a significant impact on both active and passive immuno-prophylaxis failure [15,20,29,34,39,40,65]. Studies have shown that a pregnant woman’s viral load above 10^6^ UI/mL was associated with failed neonatal prophylaxis compared to low numbers of HBV-DNA copies, which are linked to 100% success rates of active and passive infant immunization [20,39,40,65]. If the mother is HBeAg-positive, in the presence of high viral load, risk of mother-to-child HBV transmission increases [20,39,40,61,64,65].

#### 5.1.3. HBV Mutant Variants

These “escape” HBV variants have been identified in subjects with neutralizing antibodies and active HBV infection [20,49]. Infants born to mothers who carry the “wild-type” HBV usually develop chronic infection, more so in the presence of HBeAg [9,20,61]. A paper stated that precore mutants carried by chronic-HBV mothers do not correlate with increased risk of HBIG prophylaxis failure or liver disease severity over long-term follow-up [66]. A paper by Yin et al. concluded that there were no significant differences in mutation frequencies of the HBV pre-S/S gene between case and control groups, therefore, no association between pre-S/S mutations and vaccination failure could be made [67].

#### 5.1.4. Delivery Type

Current data regarding type of delivery and HBV MTCT is inconsistent. There are studies which support cesarean section in mothers with high viral load and HBeAg as a method to reduce vertical transmission [15,29,68]. The delivery method should follow obstetrical recommendations [29]. A meta-analysis from 2019 found no significant differences between type of delivery and reduced HBV MTCT [69]. Another meta-analysis from China, published in 2017, which analyzed 28 articles containing 30 databases and encompassed 9906 subjects, determined that cesarean section could reduce the risk of HBV MTCT in comparison with vaginal delivery [70]. Hou Jin et al. stated in their paper regarding algorithms for interrupting mother-to-child HBV transmission that mode of delivery should follow the usual obstetric indications and that routine c-section is not recommended for prevention of HBV MTCT [63].

#### 5.1.5. HIV Co-Infection

As with HCV-infected pregnant women, co-infection with HIV has been determined as an important risk factor for HBV MTCT [20,71]. A study in South Africa by Chasela et al. from 2014 reported that HBV VT in HIV/HBV-co-infected mothers who were also HBeAg-positive and had high HBV viral load (10^7^–10^8^ copies/mL) was 28% [71].

#### 5.1.6. HBV Genotype

There are ten main HBV genotypes—from A to J. The HBV genotype may be associated with a higher rate of viral vertical transmission [9,15,20,22]. In their review paper entitled “Vertical Transmission of Hepatitis B Virus—An Update” Fillipo Villa et al. mention that HBV MTCT is higher in East Asia, particularly in China where genotypes B and C are prevalent and most pregnant women are HBeAg+, compared to sub-Saharan Africa where genotypes A1 and E are prevalent and seroconversion to anti-HBe occurs long before gestational age [20].

A significant matter is the risk of MTCT depending on the specific time the mother contracted HBV infection during pregnancy: 60% of pregnant women who acquire acute HBV infection at or near delivery will transmit the virus to their offspring [72].

### 5.2. Risk Factors for HCV Vertical Transmission

**Figure 7:**
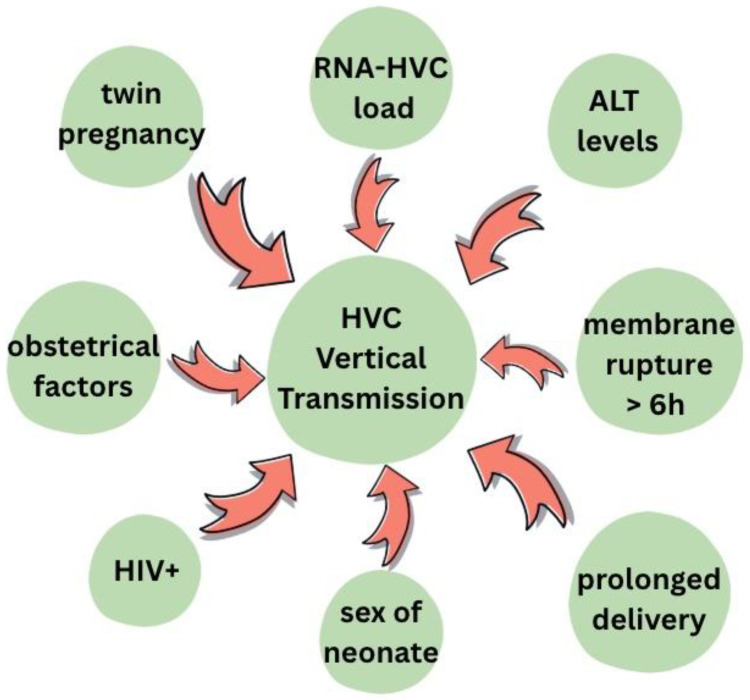
Risk factors for mother-to-child HCV transmission [10,26,32].

There are a variety of factors that may increase the risk of HCV MTCT, such as delivery method, viral load or HIV co-infection [10,26,30,32,42] (Figure 7).

#### 5.2.1. High Maternal Serum Viral Load

Elevated number of HCV copies is considered the most important risk factor for MTCT, but no clear level for viral load has been defined as a VT predictor [7,32,54,73]. Risk of VT increases proportionally with the increase in viral load above 100 UI/mL [26]. Transmission to the fetus is more likely if the mother has high HCV-RNA levels at the time of delivery [7,10,42,44,46]. Furthermore, infants who are infected with HCV genotype 1 have a higher rate of chronicity [10,27,46]. Recent studies have indicated a beneficial relationship between IL28B and spontaneous viral clearance, meaning no impact on HCV MTCT but high chance of HCV clearance in the newborn [10,27,30,46].

#### 5.2.2. Serum Levels of ALT

An elevated level of alanine aminotransferase close to delivery time or within 12 months before pregnancy reflects high viral replication and is considered an important risk factor for HCV vertical transmission [26,27,32,46].

#### 5.2.3. Prolonged Membrane Rupture, Prolonged Delivery and Obstetrical Procedures

Prolonged rupture of membranes (ROM) for more than 6 h increases risk of HCV VT, so the second stage of labor must be shortened [10,27,42,46]. The European Pediatric Hepatitis Virus C Network estimated that, for every hour of ROM, the risk of MTCT goes up by 3% [32]. The average ROM time for infected infants is 4.5 h [32].

Invasive monitoring of the fetus during delivery with scalp electrodes increases the risk of HCV MTCT, so recommendations are to avoid internal fetal monitoring for HCV+ mothers [10,32,42].

Amniocentesis is another procedure which increases risk of HCV VT [7,32,42], as the virus can be detected in amniotic fluid samples from up to 6.3% of infected mothers [32].

Episiotomy increases risk of HCV MTCT from 1.7% to 3.8% [7,32,42] because it creates a vaginal or perineal laceration, thus exposing the fetus to infected maternal blood [10,44].

#### 5.2.4. Gender of Neonate

Sex of the neonate has been reported to affect the perinatal transmission rate of HCV, as well as HBV. While male infants are more commonly infected with HBV, female infants are twice as likely to be infected with HCV, probably due to their hormonal status or genetic response to infection [26,32,44].

#### 5.2.5. HIV Co-Infection

Maternal co-infection with HCV and HIV is associated with higher rates of vertical transmission [10,26,32,42]. Co-infected pregnant women have a 3 to 4 times higher risk of transmitting infection to their offspring, with a VT rate ranging from 8.7% to 19% [10,32]. Seroprevalence of HCV in HIV-infected pregnant women is between 17% and 57% [10]. A meta-analysis found that HIV-HCV co-infection increases the risk of perinatal HCV transmission by 90% [26]. Even with antiretroviral therapy, the risk for HVC VT remains high [10,26,42].

#### 5.2.6. Twin Pregnancies

Twin pregnancies discordant for HCV MTCT are yet another supporting factor [26]. Studies suggest that the second twin might be at higher risk of infection, potentially because of prolonged exposure to infected maternal blood during birth. Even in twins that share the same amniotic sac, HCV transmission might happen only in one of them, suggesting involvement of factors beyond the intrauterine environment [26,27,74].

HLA classes I and II are ideal candidate genes to study associations between HCV infection and outcomes, as each HLA has an important role in the immune response against HCV [6,27]. A multicenter study from 2019 investigated the connection between HLA-DRB1 alleles and HCV infection outcomes among 162 Egyptian families. Results showed that carrying HLA-DRB1*030101 and DRB1*130101 alleles was associated with the risk of progression to chronic HCV infection. Those carrying DRB1*040101, DRB1*040501, DRB1*7:01:01 and DRB1*110101 alleles were protected against HCV infection [6].

Regarding twin pregnancies, no consistent relation was observed between the presence or absence of HCV infection in the first versus second infant, even in identical twins [26,74].

## 6. Prevention of Vertical Transmission

### 6.1. Management Strategies for Prevention of HBV VT During Pregnancy

The first step to reduce global burden of chronic HBV infection, especially in endemic areas, is to prevent mother-to-child transmission. The key to obtaining this goal is through rigorous screening and prophylactic methods applied both during pregnancy and after delivery. Two main preventive arms have been developed to lower HBV MTCT—antiviral therapy for pregnant women and immuno-prophylaxis in infants [20,21,24,29,64,65].

#### 6.1.1. Maternal Screening

**Figure 8:**
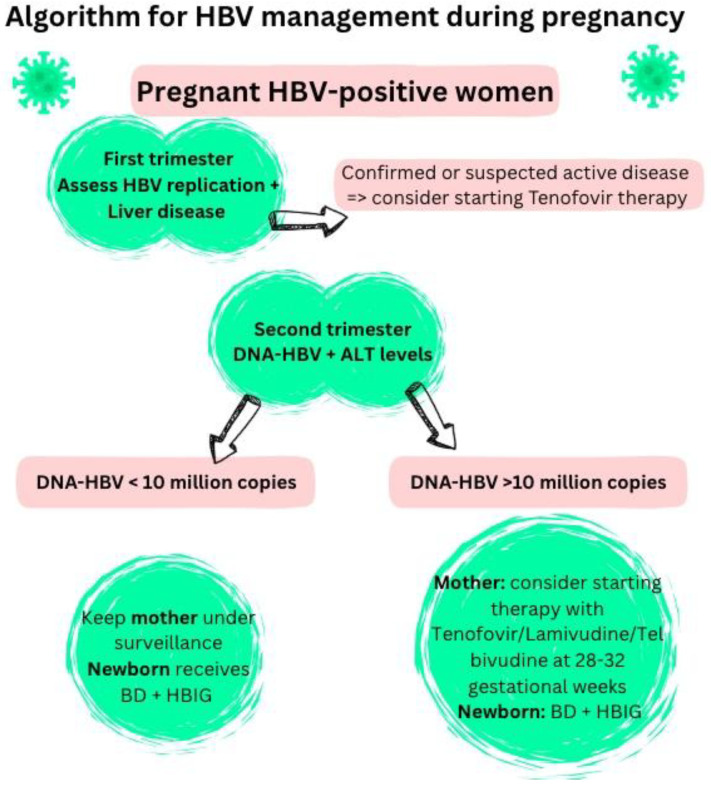
Management of HBV infection during pregnancy [21,24,29,80,81].

Step one for prevention is screening—all pregnant women should be tested for HBsAg status. If HBsAg is detected, the next step is searching for HBeAg and HBV-DNA, as well as contacting a specialist in infectious diseases (Figure 8). The mother should also be further investigated with hepatitis B core antibody, hepatitis B e antibody, serum aminotransferase levels and liver imaging. In the last few years other viral markers have been proposed for evaluation—covalently closed circular DNA (cccDNA) and circulating HBV-DNA [9,21,24,29,63,75,76,77,78,79].

If a pregnant woman is HBsAg-negative at first testing but comes from a high-risk family environment (infected partner or family members, risky habits), a second serological evaluation should be performed before delivery [24,35,80,81,82].

#### 6.1.2. Hepatitis B Vaccine During Pregnancy

**Figure 9:**
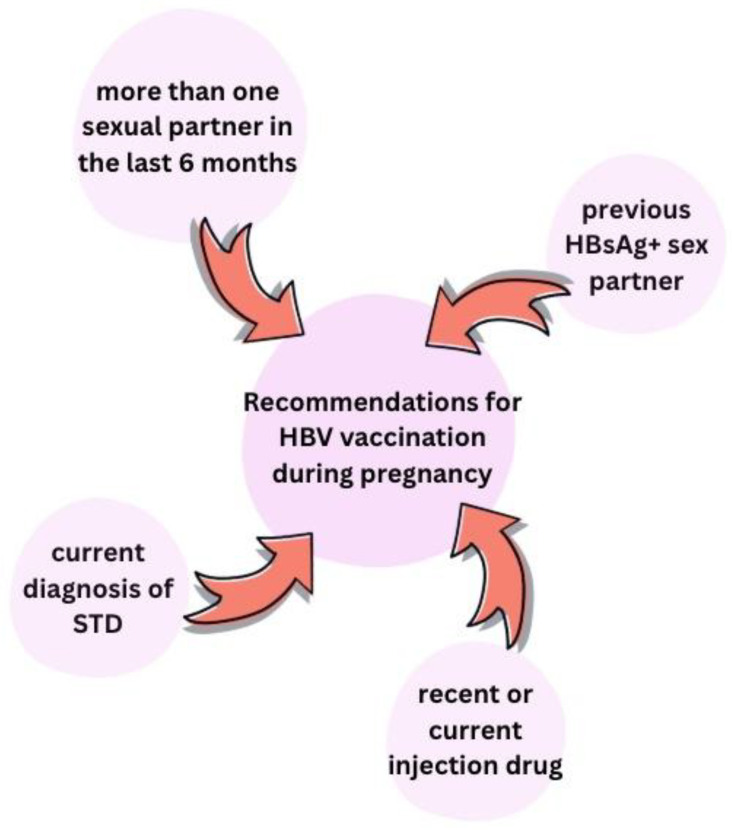
Risk factors to support HBV vaccination during pregnancy [10,24,49,52]. STD—sexual transmitted disease.

Vaccination against HBV in a pregnant woman is considered to be safe and effective. It is recommended to women not immune to or not infected with HBV with or without risk factors, such as more than one sexual partner or a previous partner with HBsAg+, IV drug use or recent diagnosis of an STD (Figure 9). Maternal antibodies produced afterwards cross the placenta and enter the fetus’s circulation, offering protection. But active vaccination of the newborn should be further pursued because maternal antibody titer rapidly fades after birth [10,24,35,37,49,52].

#### 6.1.3. Hepatitis B Immunoglobulin (HBIG) During Pregnancy

Approximately 10% of infants born to HBV-infected mothers, despite rigorous administration of HBIG and HBV vaccine immediately after birth, develop long-term HBV infection. This is why additional prophylaxis methods during pregnancy should be taken into consideration, such as giving the mother purified HBIG. In terms of real efficacy, doses and route of administration, studies do not offer exact recommendations [24,35,52,75,83,84].

Veronese et al. mentioned a Cochrane study including more than 6000 pregnant women who received 200 UI of HBIG in the second and third trimesters; the review found low-quality evidence about the impact of HBIG on HBsAg and HBV-DNA status of newborns [24].

Despite maternal–fetal diffusion of antibodies, after HBIG therapy for pregnant women, being maximal during the third trimester, Eke et al. concluded there was little evidence regarding the benefits of antenatal administration to HBV mothers compared with no intervention. Newborn outcomes, such as HBV-DNA, HBsAg or HBeAg, were not significantly improved [52].

But a paper regarding cost-effectiveness of maternal therapy to prevent HBV MTCT, classified as “level III of confidence”, concluded that, for HBIG administered in the third trimester, 9.5 cases of chronic hepatitis B virus infections were prevented for every 100 pregnant women treated, with a cost savings of USD 5887 [2].

**Figure 10:**
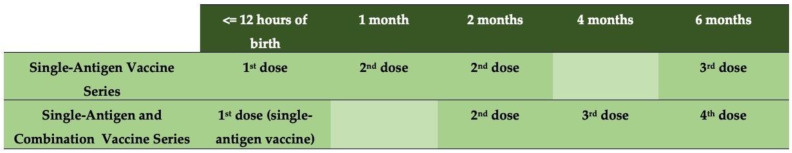
Immunization algorithm for newborns >2000 g birth weight [79].

The CDC proposes, for pediatricians, a useful and easy-to-apply algorithm for the management of infants born to women with HBV infection, depending on birth weight. For newborns above 2000 g (Figure 10), BD vaccination and HBIG should be administered no later than 12 h after birth in separate limbs. The vaccine series must be completed with two additional doses of single-antigen vaccine (three doses in total) or with three additional doses of combined vaccine (four doses in total). The last dose should be administered after 6 months of age [79].

**Figure 11:**
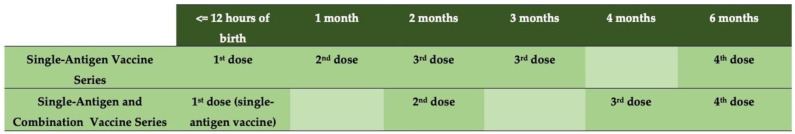
Immunization algorithm for newborns <2000 g birth weight [79].

For newborns with birth weight under 2000 g (Figure 11), BD vaccination and HBIG should be administered within the first 12 h after birth in separate limbs. Then, the vaccination scheme must be completed with three additional single-antigen or combined vaccine doses (four doses in total). The final dose should be administered no earlier than 6 months of age [79].

#### 6.1.4. Antiviral Therapy During Pregnancy

There are several papers which describe the use of nucleotides or nucleoside analogues during the third trimester in pregnant women with high viral load and HBeAg, alongside newborn HBIG and HBV vaccine administration, as an effective method to reduce vertical transmission. AASLD and APSAL recommend, for all high-load HBV-DNA mothers, to combine active and passive infant immunization with antiviral therapy starting at 28–32 weeks of gestation for high effectiveness of MTCT prevention strategies. In terms of single versus combined antiviral therapy, there is no evidence base during pregnancy to date [1,35,36,56,58,59,85,86,87,88].

Antiviral therapy during pregnancy is widely accepted because it lowers the mother’s HBV-DNA level and therefore plays a significant positive role in eliminating HBV MTCT [20,24,29,35,36,37,39,85]. Antiviral therapy is generally safe to use in pregnant women, but there is a risk of hepatitis flare after treatment is stopped postpartum, meaning moderate increases in ALT level [1,35,56,58,59,86,87,88]. However, there have been cases with progression to liver failure, more so if women were younger and HBeAg-positive [35].

An indication for receiving antiviral therapy during pregnancy is elevated viral load in the second trimester (12–24 gestational weeks). If quantitative testing is unavailable, HBeAg must be used as an alternative serological marker for viral replication [20,24,29,37,56,58,59,86].

The appropriate time to begin antiretroviral therapy during pregnancy is still under debate. Studies have suggested that an earlier start might have additional benefits in preventing placental infection and IUT [24,58,59].

When it comes to which antiretroviral drug to use in a pregnant woman, there are three main choices considered to be safe and effective, each with their pros and cons—lamivudine, telbivudine and tenofovir disoproxil fumarate (TDF) [20,24,29,36,37,39,88]. Tenofovir alafenamide fumarate (TAF) is a prodrug of tenofovir that can be used in a lower dose, thus producing lower circulating drug levels in pregnant women [1,24,59,88]. Entecavir is not recommended during pregnancy due to its questionable use [20,59].

Lamivudine is the oldest antiviral drug used to treat chronic B hepatitis in pregnant women [85], but it has a low genetic barrier to resistance [24], therefore it has some limitations because of poor antiviral effect in the third trimester [20,24,36,37,59].

Compared to lamivudine, telbivudine is a newer antiviral agent, with higher efficacy in preventing HBV VT [37,56,58,59,87], but which also has a low genetic barrier to resistance [24,59,87]. A prospective, open-labeled study from 2018, which assessed the efficacy of telbivudine in highly viremic mothers (>5 log10 UI/mL) and included 91 subjects in the treatment group, concluded that the drug was effective in reducing HBV MTCT—the rate was 0% in the treatment group and 9.5% in the observational group (*p* = 0.042) [56].

Tenofovir is the treatment of choice for HBV-positive mothers because of its high antiviral activity, high barrier to resistance and safety profile [20,24,37,39,86,89]. It has also been used to safely treat pregnant women with HIV infection [37,86,89]. An HBV MTCT prevention protocol for a one-arm, open-labeled intervention, by Bierhoff et al., proposes TDF during pregnancy as a potential alternative to HBIG, which is far more expensive and difficult to obtain in limited-resource settings [86]. This study from 2020 aimed to gather pregnant women under 20 weeks of gestation from Thailand and Myanmar and investigate if TDF administered to mothers alongside standard immunization of newborns (HBIG and HBV vaccination) could result in better VT prevention [86]. Another review paper by Pan QC about the role of TDF in preventing MTCT which searched the literature from 2015 to 2021 has concluded that therapy initiated from the second or third trimester for highly viremic mothers is the key to success. Furthermore, if TDF was started earlier (second trimester), a better control of viremia was achieved [89]. Table 3 summarizes the indications of TDF therapy in HBV-DNA-positive pregnant women [1,24,29,36,37,59,86,89].

A systematic review and meta-analysis of almost 7000 papers including more than 70,000 HBV+ mothers concluded that approximately 20% of infected pregnant women are eligible for prenatal antiviral prophylaxis (PAP) [90]. The risk of child infection, when an infant is born to a high-viremia and HBeAg-positive mother, following birth dose vaccination without HBIG and PAP was almost 15% [90]. The risk of child infection, in an infant born to a low-viremia and HBeAg-negative mother, following birth dose vaccination without HBIG and PAP was 0.14% [90]. This meta-analysis is essentially supporting treatment recommendations from Table 3—low viral load and negative status of HBeAg are not indicators for antiviral therapy during pregnancy. In these cases, standard active and passive newborn immunization should be pursued.

Regarding the question about when it would be the right time to stop antiretroviral therapy, Veronese et al. gathered in their paper recommendations from five international profile societies [24]. These recommendations about the appropriate time to stop antiviral therapy after delivery are summarized in Table 4.

The time to discontinue antiviral therapy for pregnant women depends on the initial purpose of use. If drugs were recommended for prevention of HBV MTCT, after delivery, serological markers should be quantified—if the mother is HBsAg-negative and HBeAg-negative, then antiviral therapy should be interrupted. If this is not the case, and serological status still indicates active disease, the mother should be further treated after delivery. There is the discussion of potential maternal hepatitis flare after ending antiviral treatment, but most cases are asymptomatic with low to moderate increases in ALT level [20,24,29,37,58,85,86,87].

Regarding safety of breastfeeding in mothers who received or are still under antiviral therapy, evidence to date is still debatable [20,37,59]. EASL maintains that the safety profile of antiretrovirals in breastfeeding women is uncertain, but the WHO recommends that HIV-positive mothers under antiretroviral therapy should continue to breastfeed [37,76]. Furthermore, the odds of fetus IUT after exposure to antivirals are higher than through breast milk [58,87]. Di Fillipo Villa et al. mention in their paper a study about pregnant women treated with lamivudine—total concentration of the drug was almost 50% lower in breast milk compared to blood (684 ng/mL versus 1070 ng/mL) [20]. Therefore, antiviral therapy should be considered after delivery in breastfeeding mothers to eliminate residual transmission [20,29,35,37,87].

### 6.2. Management Strategies for Prevention of HBV VT at Birth

A single-center retrospective study (2014–2019) from a North Carolina Hospital stated that 98% of followed neonates, born to HBV-infected mothers, received the AASLD-recommended prophylaxis dyad (HBIG + HBV vaccination) within 12 h after birth. The paper stated that AASLD guidelines should be revised to include HBeAg-positive mothers in the risk group or to lower the cut-off for viral load to <200,000 IU/mL, because 62% of pregnant women in this study were HBeAg+ or had low viremia with HBeAg [38]. Administration of HBIG and HBV vaccine prevents in most cases perinatal viral transmission [47,78,91,92,93].

An analysis conducted in Namibia by CR Tamandjou Tchuem et al. about cost-effectiveness of different types of HBV MTCT prevention methods proposed a four-step interventional strategy to eliminate risk of HBV infection in offspring (step 1: universal BD vaccination, step 2: universal BD vaccination + targeted HBIG, step 3: universal BD vaccination + HBV viral load testing + maternal antiviral prophylaxis + targeted HBIG, step 4: universal BD vaccination + HBeAg testing + maternal antiviral prophylaxis + targeted HBIG) (Table 5) [80]. This analysis determined that the current intervention against HBV MTCT in Namibia, which includes birth dose vaccination and targeted HBIG, is cost-effective, but elimination of VT can be achieved only by using PAP in combination. Rate of MTCT from HBeAg-negative mothers was 1.02% in newborns who received only BD vaccination, compared to 0.34% in newborns with timely BD and HBIG [80]. When the mother was HBeAg-positive, rate of MTCT for newborns who received only timely BD vaccination was 24.68%, but if HBIG was added to the prophylaxis intervention, the percentage went down to 6.9% [80]. In newborns with both BD vaccination and HBIG, from HBeAg-positive mothers who received TDF therapy before birth, rate of MTCT was low (0.73%) [80].

In another review paper regarding HBV VT in sub-Saharan Africa including 60 studies, the most cost-effective intervention to reduce HBV MTCT was timely BD vaccination followed by three-dose infant vaccine series [81]. A meta-analysis by Machaira et al. comparing two prophylaxis strategies (HBV vaccination alone versus HBV vaccine + HBIG) in neonates born to HBsAg+/HBeAg- mothers concluded that the vaccine alone seemed to be equally effective in preventing HBV MTCT as the combination [64]. In a position paper by Stevens et al. regarding eradication of HBV and the critical role of preventing perinatal transmission, the authors stated that the vaccine alone at birth or within one week of birth, with an efficacy of 70–80% and 90% coverage, still predicts a residual carrier rate in children of 1% due to perinatal transmission [65]. In contrast with the result of the previous paper (by Machaira et al.), this one implies that the birth dose vaccine alone is not sufficient for ceasing HBV VT, especially in HBeAg-positive mothers [65]. To support this statement, a recent study by Jourdain et al. reported that prompt administration of HBIG within 4 h after birth, alongside an increase in HBV vaccination number (0–1–2–4–6 months), resulted in very low rates of perinatal transmission (2%) [1].

Table 6 carefully summarizes recommendations for neonatal anti-HBV immuno-prophylaxis, respecting variations such as single versus combined prevention approaches, HBsAg status of pregnant women, infant’s birth weight, immunization of newborns in critical conditions and approaches in the case of delayed vaccination [8,24,29,47,76,79,80,93,94].

### 6.3. Management Strategies for Prevention of HCV VT

Vertical transmission in the case of HCV-positive mothers depends on the viral load—levels above 6 log UI/mL are associated with high risk of HCV MTCT [7,27,73]. There are a few published cases reporting safety of antiviral therapy during pregnancy [7,25,85]. One trial with ledipasvir/sofosbuvir is limited to a phase 1 status, with 100% cure and no safety concerns [85]. However, these INF-free and ribavirin-free regimens, that might represent an exciting prospect, cannot be used yet during pregnancy because of potential toxic effects on the fetus [25]. It is widely known that ribavirin, due to its teratogenic traits which last up to 6 months after treatment cessation, is contraindicated during pregnancy [85].

Clinicians are in deep need of a composite score which will identify populations of pregnant women at high risk for HCV MTCT. This predictive score would allow them to decide which mother-to-be should benefit from antiviral therapy. A multicenter study by Wasuwanich et al. proposed the following statement: low HCV-RNA load under 6 log UI/mL has an excellent negative predictive value [7]. Pregnant women with low viral load are at low risk for HCV VT, thus no antiviral therapy during pregnancy is required [7]. For those with high viral load (>6 log UI/mL), antiviral therapy might be beneficial [7]. When it comes to HCV, there is a huge lack of preventive MTCT measures, but this is no contraindication for a future pregnancy, more so because of what is known as “spontaneous viral clearance” (SVC). This term refers to those children infected with HCV via vertical transmission who have cleared circulating HCV-RNA and associated biochemical remission of hepatitis [25].

All infants born to HCV-positive mothers, regardless of maternal HCV-RNA load, must be tested for HCV infection and followed up a least until 18 months of age (Table 7) [7,25,43,44,77,95]. Epstein et al. proposes a well-structured algorithm for HCV infection diagnosis of children born to seropositive mothers [44]. According to a large cohort study of more than 1700 children, 3.9% of infants born to HVC-RNA-positive mothers remain infected at the age of 5 years [46].

## 7. Assessment of Infants Born to Infected Mothers

Actively searching for vaccine response and infection status is recommended in all offspring of HBV- and HCV-positive mothers. Follow-ups of these newborns are mandatory for their health and development, thus ensuring timely interventions and support in affected families. These infants are at an increased risk of acquiring the infection, which possibly leads to chronic liver disease, and that is why early detection and management can significantly improve outcomes for these children. Follow-up during the breastfeeding period is also important, especially for those infants who did not receive timely BD vaccination [77,79,95,96,97].

### 7.1. Assessment of Newborns from HBV-Positive Mothers

**Figure 12:**
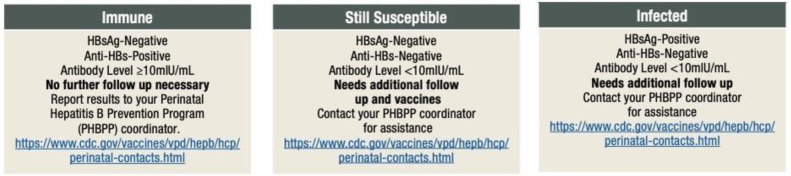
Interpreting postvaccination serologic test results in infants born to HBV-positive mothers [79].

Testing exposed infants is quite problematic. Searching for HBsAg and anti-HBs antibodies should be performed first at 1–2 months of age. If the initial test indicates that the infant is HBsAg-positive, further evaluation and follow-up care will be necessary to monitor liver function and manage any potential complications. Within the first six months of life, HBsAg may be inconsistently detectable in infected infants. For an accurate result, postvaccination serologic testing (for those who received BD vaccination) should be performed again at 9–12 months of age. This includes checking status of HBsAg and anti-HBs antibodies (Figure 12). If both are negative, the infant did not develop an adequate immune response and further additional dosages may be needed. An infant who has negative HBsAg and high levels of anti-HBs antibodies is well protected. Acquired infection is diagnosed if HBsAg is persistent after 6 months or more [77,78,79,93,96]. PVST should not be performed earlier than 9 months of age because antibodies from the after-birth HBIG injection may interfere with test results [63,93]. Recommendations are not to test for anti-HBc antibodies due to false-positive results because of passively acquired maternal antibodies [92,96].

### 7.2. Assessment of Newborns from HCV-Positive Mothers

Approximately 6–7% of perinatally exposed children will acquire perinatal HCV infection [95]. The CDC strongly recommends perinatal testing for hepatitis C among infants born to positive mothers for an early diagnosis [95]. Infected children must be detected so they can have access to curative treatment before developing clinical manifestations and complications from chronic hepatitis C [77,95].

HCV-RNA should be searched for in all perinatally exposed infants at or after 2 months of age (until 6 months) to identify who stands at risk to develop chronic HCV infection. Infants with undetectable HCV-RNA do not require further investigations. Those with detectable HCV-RNA at 2–6 months of age (lower limit of detection being 15 IU/mL) are considered infected, because one positive result is sufficient to determine perinatal infection [77,95,97,98].

Because maternal antibodies, passively passed through the placenta, remain in the child’s bloodstream up until 18 months of age, testing for anti-HCV antibodies in infants has no indication. Infants and children aged 7–17 months, who were perinatally exposed to HCV and have not been previously tested, should be searched for HCV-RNA. Those aged above 18 months can be tested for anti-HCV antibodies, alongside HCV-RNA [77,95,97].

## 8. Future Directions

This systematic review has its limitations. The search strategy may not have captured all relevant studies, as only a limited database was utilized, thus this narrow search strategy could have missed relevant studies, alongside a potential bias in the selected literature. Furthermore, the findings of this review are established upon the quality of the original studies. Therefore, the reliability of the collective evidence is constrained by the methodological rigor of each individual study.

Global health sector strategy calls for the elimination of viral hepatitis as a public health threat by 2030. The goal is to reduce new infections by 90% and mortality by 65%. The number of deaths from chronic liver disease and primary liver cancer due to viral hepatitis is increasing over time, while mortality caused by tuberculosis and HIV is declining [17].

Future directions in preventing hepatitis B and C in newborns focus on improving vaccination strategies, screening and maternal interventions.

Universal newborn vaccination against hepatitis B, combined with timely administration of hepatitis B immunoglobulin, has proven highly effective in preventing mother-to-child transmission and efforts are ongoing to ensure full coverage worldwide. Advances in maternal screening during pregnancy are aiding early identification of infected mothers, enabling targeted interventions to reduce vertical transmission [93,99,100,101].

For hepatitis C, education campaigns aim to raise awareness about the importance of prenatal screening and vaccination. Innovative approaches, such as maternal antiviral therapy and improved diagnostic tools, hold promise for further decreasing the incidence of hepatitis C in newborns, ultimately contributing to the goal of elimination [8,17].

A prospective study regarding an infant vaccination program against hepatitis B in Taiwan showed that seroprevalence of HBsAg decreased from 9.8% (before 1984) to 1% (25 years later). The epidemiological study surveyed the Taipei metropolitan area every 5 years from 1984 to 2014. It showed that 6.7% of individuals born before universal vaccination were found positive for HBsAg, compared to 0.5% of those born afterwards [102].

Another prospective study regarding seroprevalence of hepatitis B in the general population of Shandong Province in eastern China, 30 years after implementing a national vaccination program, concluded that, through routine vaccination of newborns, substantial control of HBV infection was achieved. The overall prevalence of HBsAg in 2023 was 2.25% and, in individuals younger than 30 years of age, prevalence dropped to 0.28%. The highest HBsAg prevalence (5.63%) was found among older people (>50 years old), concluding that the vaccination program has successfully fulfilled its mission [101].

These studies are highly relevant because they demonstrate, with clear numbers, how important vaccination of newborns is, especially in high-risk populations. Such studies have a major impact on public health policies, strengthening the need for global immunization programs.

## 9. Conclusions

Vertical transmission of hepatitis B and C continues to be a major contributor to the global burden of chronic viral hepatitis. Despite advances in screening and prevention measures, mother-to-child transmission remains a critical pathway for new infections, particularly in areas with high endemic status.

For hepatitis B, the combination of maternal antiviral therapy during pregnancy and timely administration of the HBV vaccine alongside HBIG to the newborn has proven to be highly effective in lowering transmission rates. However, challenges such as late maternal diagnosis, areas with limited access to health care and vaccine coverage gaps still interfere with the efforts of eradicating HBV mother-to-child transmission.

In contrast, hepatitis C vertical transmission lacks an effective vaccine or immuno-prophylaxis, turning prevention strategies into a continuous battle. Current approaches focus on identifying infected mothers through screening and considering antiviral treatment options during pregnancy, although safety and efficacy are yet to be established. Risk factors impacting rates of transmission, like maternal viral load or co-infections, underscore the need for individualized care in the case of HCV-positive women.

Overall, strengthening prenatal care programs, improving access to diagnostic and therapeutic resources and enhancing public health policies are essential to curb vertical transmission of both hepatitis B and C. Continuous research into novel prophylaxis and treatment methods, alongside education and awareness campaigns, will be pivotal in reaching the ultimate goal—eliminating mother-to-child transmission and reducing the long-term health consequences for affected children.

## Figures and Tables

**Table 1:** Serologic and molecular markers in hepatitis B infection [9,10,17,20].

Serologic and Molecular Markers	Significance	Vertical Transmission Risk
HBsAg+	First infection marker Positive after 6 months means chronic infection	Very high
Anti-HBs antibodies	After vaccination or clinical resolution of acute infection	Low
HBeAg+	Elevated viral replication Related to immune tolerance phase	Very high
Anti-HBe antibodies	Indicates resolution of infection if it is associated with anti-HBs+	Low
IgM Anti-HBc antibodies	Indicates acute or recent infection Can reappear during chronic infection reactivation (pregnancy)	Very high
HBV-DNA	Indicates viral replication	Depends on the viral load

**Table 2:** Management recommendations for pregnant women with active HBV infection [15,29,34].

Infection Markers	Management Approach
HBV-DNA+ ALT ≥ 5 × ULN	Requires further examination (abdominal ultrasound, fibrosis score) and antiviral therapy as primary recommendation
HBV-DNA+ ALT ≥ 1 and ≤ 5 × ULN and Total bilirubin ≤ 2 × ULN	“Wait and watch” approach until 24 weeks of gestation If levels remain the same, antiviral therapy should be administered
HBV-DNA+ ALT ≥ 5 × ULN or Total bilirubin ≥ 2 × ULN	Requires further examination (abdominal ultrasound, fibrosis score) and antiviral therapy as primary recommendation
HBV-DNA+ Normal levels for ALT No manifestations of liver cirrhosis	“Wait and watch” approach until 24 weeks of gestation If ALT ≥ 1× ULN at follow-up, further testing required and, according to ALT value, consider antiviral therapy
Undetectable levels	Test again for HBV-DNA levels at 24 weeks of gestation

ULN—upper limit of normal value.

**Table 3:** Recommendations for tenofovir disoproxil fumarate in HBV viremic mothers [1,29,59,86,89].

HBV-DNA Level	TDF Therapy	Alternative Drugs
>2 × 10^5^ UI/mL	Start at 28 weeks of gestation	TAF or telbivudine if mother has osteoporosis, kidney damage, severe GI symptoms
<2 × 10^5^ UI/mL	NOT recommended (perform standard active and passive immunization of newborn)	NOT recommended
≥2 × 10^5^ UI/mL First follow-up after 28 weeks of gestation	Immediate initiation of TDF	TAF or telbivudine if mother has osteoporosis, kidney damage, severe GI symptoms

GI = gastrointestinal tract.

**Table 4:** Recommendations for cessation of antiviral therapy in HBV+ mothers [24].

Societies	Time for Treatment Cessation
AASLD	At birth to 3 months
EASL	Up to 3 months after delivery
APASL	At delivery
NIHCE	1 to 3 months after delivery
CMA	At delivery

AASLD = American Association for the Study of Liver Diseases, EASL = European Association for the Study of the Liver, APASL = Asian Pacific Association for the Study of the Liver, CMA = Chinese Medical Association, NIHCE = National Institute for Health and Care Excellence.

**Table 5:** Interventional strategy for HBV MTCT prevention in Namibia (summary) [80].

Strategy Type	Intervention
Strategy 1 ⮚ Universal BD vaccine	⮚ No prenatal HBV screening ⮚ No PAP
Strategy 2 ⮚ Universal BD vaccine ⮚ Targeted HBIG	⮚ HBsAg as prenatal screening ⮚ No PAP ⮚ HBIG + BD vaccine for HBV-exposed neonates ⮚ BD vaccine for HBV-unexposed neonates
Strategy 3 ⮚ Universal BD vaccine ⮚ Test HBV-DNA level ⮚ PAP ⮚ Targeted HBIG	⮚ HBsAg as prenatal screening ⮚ Test HBV-DNA level in HBsAg+ mothers ⮚ PAP for high viral load mothers ⮚ HBIG + BD vaccine for HBV-exposed neonates ⮚ BD vaccine for HBV-unexposed neonates
Strategy 4 ⮚ Universal BD vaccine ⮚ Test HBeAg status ⮚ PAP ⮚ Targeted HBIG	⮚ HBsAg as prenatal screening ⮚ Test HBeAg in HBsAg+ mothers ⮚ PAP for HBeAg+ mothers ⮚ HBIG + BD vaccine for HBV-exposed neonates ⮚ BD vaccine for HBV-unexposed neonates

PAP = prenatal antiviral therapy, BD = birth dose HBV vaccination.

**Table 6:** Recommendations for newborn anti-HBV prophylaxis [8,24,29,47,76,79,80,93,94].

Newborn’s General Status and Mother’s HBsAg Status	General Recommendations
Routine vaccination	⮚ Recombinant yeast HBV vaccine 10 mcg/0.5 mL (anterior lateral deltoid or thigh muscle) within 12 h of birth ⮚ HBIG 100 UI (corresponding contralateral muscle) within 12 h of birth ⮚ Recombinant yeast HBV vaccine 10 mcg/0.5 mL at 1 month and 6 months
Normal newborn HBsAg-positive mother	⮚ Recombinant yeast HBV vaccine 10 mcg/0.5 mL within 12 h of birth ⮚ HBIG 100 UI within 12 h of birth ⮚ Recombinant yeast HBV vaccine 10 mcg/0.5 mL at 1 month and 6 months
Normal newborn Mother’s HBsAg status unknown	⮚ Must be treated the same as newborns with HBsAg-positive mother
Normal newborn HBsAg-negative mother	⮚ Recombinant yeast HBV vaccine 10 mcg/0.5 mL (anterior lateral deltoid or thigh muscle) within 12 h of birth ⮚ Recombinant yeast HBV vaccine 10 mcg/0.5 mL at 1 month and 6 months
Low-birth-weight newborn (under 2000 g) Preterm newborn (under 37 weeks of gestation) HBsAg-positive mother	⮚ Recombinant yeast HBV vaccine 10 mcg/0.5 mL (anterior lateral deltoid or thigh muscle) within 12 h of birth ⮚ HBIG 100 UI (corresponding contralateral muscle) within 12 h of birth ⮚ Recombinant yeast HBV vaccine 10 mcg/0.5 mL at 1 month, 2 months and 7 months
Low-birth-weight newborn (under 2000 g) Preterm newborn (under 37 weeks of gestation) Mother’s HBsAg status unknown	⮚ Must be treated the same as newborns with HBsAg-positive mother
Low-birth-weight newborn (under 2000 g) Preterm newborn (under 37 weeks of gestation) HBsAg-negative mother	⮚ Recombinant yeast HBV vaccine 10 mcg/0.5 mL (anterior lateral deltoid or thigh muscle) within 12 h of birth ⮚ Recombinant yeast HBV vaccine 10 mcg/0.5 mL at 1 month, 2 months and 7 months
Very low birth weight (under 1500 g) Severe birth defects Neonatal hypoxia Respiratory distress syndrome HBsAg-positive mother	⮚ Recombinant yeast HBV vaccine 10 mcg/0.5 mL (anterior lateral deltoid or thigh muscle) within 12 h of birth ⮚ HBIG 100 UI (corresponding contralateral muscle) within 12 h of birth ⮚ Recombinant yeast HBV vaccine 10 mcg/0.5 mL at 1 month, 2 months and 7 months As soon as possible after stabilizing vital signs
Very low birth weight (under 1500 g) Severe birth defects Neonatal hypoxia Respiratory distress syndrome Mother’s HBsAg status unknown	⮚ Must be treated the same as newborns with HBsAg-positive mother
Very low birth weight (under 1500 g) Severe birth defects Neonatal hypoxia Respiratory distress syndrome HBsAg-negative mother	⮚ Recombinant yeast HBV vaccine 10 mcg/0.5 mL (anterior lateral deltoid or thigh muscle) within 12 h of birth ⮚ Recombinant yeast HBV vaccine 10 mcg/0.5 mL at 1 month, 2 months and 7 months As soon as possible after stabilizing vital signs
Delayed vaccination	⮚ Second dose of HBV vaccine can be delayed (no longer than 3 months) ⮚ Third dose of HBV vaccine can still be given at 6 months of age

**Table 7:** Screening for HVC-infected offspring [7,25,43,44,77,95].

HVC Infection Markers	HVC Infection Status
Two positive HCV-RNA samples at least 1 month apart	HVC infection
Anti-HVC antibodies at/beyond 18 months of age	HVC infection
Two negative HCV-RNA samples at least 1 month apart	Non-infected child
Negative anti-HVC antibodies at any age	Non-infected child

## Data Availability

The original contributions presented in the study are included in the article, further inquiries can be directed to the corresponding author.

## Supplementary Materials

The following supporting information can be downloaded at: https://www.mdpi.com/article/10.3390/v17101395/s1, the PRISMA Checklist.

## Abbreviations

AASLD	the American Association for the Study of Liver Diseases
ALT	alanine aminotransferase
Anti-HBc antibodies	antibodies against hepatitis B core antigen
Anti-HBe antibodies	antibodies against hepatitis B e antigen
Anti-HBs antibodies	antibodies against hepatitis B surface antigen
Anti-HCV antibodies	antibodies against hepatitis C virus
Anti-HIV antibodies	antibodies against human immunodeficiency virus
APSAL	the Asian Pacific Association for the Study of the Liver
BD	birth dose HBV vaccination
cccDNA	covalently closed circular DNA
CMA	the Chinese Medical Association
DNA	deoxyribonucleic acid
EASL	the European Association for the Study of the Liver
GI	gastrointestinal tract
HBeAg	hepatitis B e antigen
HBIG	hepatitis B immunoglobulin
HBsAg	hepatitis B surface antigen
HBV	hepatitis B virus
HBV-DNA	deoxyribonucleic acid of hepatitis B virus
HCV	hepatitis C virus
HIV	human immunodeficiency virus
HLA	human leukocyte antigen
IUT	intrauterine transmission
IV	intravenous
MTCT	mother-to-child transmission
NIHCE	the National Institute for Health and Care Excellence
PAP	prenatal antiviral prophylaxis
PVST	postvaccination serologic testing
HCV-RNA	ribonucleic acid of hepatitis C virus
STD	sexually transmitted disease
SVC	spontaneous viral clearance
TAF	tenofovir alafenamide fumarate
TDF	tenofovir disoproxil fumarate
VT	vertical transmission
WHO	World Health Organization

## References

[1] Jourdain G., Ngo-Giang-Huong N., Harrison L., Decker L., Khamduang W., Tierney C., Salvadori N., Cressey T.R., Sirirungsi W., Achalapong J. (2018). Tenofovir versus Placebo to Prevent Perinatal Transmission of Hepatitis B. N. Engl. J. Med..

[2] Unal E.R., Lazenby G.B., Lintzenich A.E., Simpson K.N., Newman R., Goetzl L. (2011). Cost-Effectiveness of Maternal Treatment to Prevent Perinatal Hepatitis B Virus Transmission. Obstet. Gynecol..

[3] Chilaka V.N., Konje J.C. (2021). Viral Hepatitis in pregnancy. Eur. J. Obstet. Gynecol. Reprod. Biol..

[4] Ruiz-Extremera Á., Díaz-Alcázar M.D.M., Muñoz-Gámez J.A., Cabrera-Lafuente M., Martín E., Arias-Llorente R.P., Carretero P., Gallo-Vallejo J.L., Romero-Narbona F., Salmerón-Ruiz M.A. (2020). Seroprevalence and epidemiology of hepatitis B and C viruses in pregnant women in Spain. Risk factors for vertical transmission. PLoS ONE.

[5] Ndzie Ondigui J.L., Mafopa Goumkwa N., Lobe C., Wandji B., Awoumou P., Voussou Djivida P., Peyonga P., Manju Atah S., Verbe V., Kamgaing Simo R. (2024). Prevalence and risk factors of transmission of hepatitis delta virus in pregnant women in the Center Region of Cameroon. PLoS ONE.

[6] El-Bendary M., Neamatallah M., Elalfy H., Besheer T., Kamel E., Mousa H., Eladl A.H., El-Setouhy M., El-Gilany A.H., El-Waseef A. (2019). HLA Class II-DRB1 Alleles with Hepatitis C Virus Infection Outcome in Egypt: A Multicentre Family-based Study. Ann. Hepatol..

[7] Wasuwanich P., So J.M., Presnell B., Karnsakul W., Egerman R.S., Wen T.S. (2024). A Composite Score for Predicting Vertical Transmission of Hepatitis C: A Multicenter Study. Pathogens.

[8] (2024). Global Hepatitis Report 2024: Action for Access in Low- and Middle-Income Countries.

[9] Belopolskaya M., Avrutin V., Kalinina O., Dmitriev A., Gusev D. (2021). Chronic hepatitis B in pregnant women: Current trends and approaches. World J. Gastroenterol..

[10] Dunkelberg J.C., Berkley E.M., Thiel K.W., Leslie K.K. (2014). Hepatitis B and C in pregnancy: A review and recommendations for care. J. Perinatol..

[11] Belete D., Fekadie E., Kassaw M., Fenta M., Jegnie A., Mulu T., Adane G., Abebe W., Amare A. (2024). Seroprevalence of hepatitis B virus and hepatitis C virus infection among pregnant women attending antenatal care at Guhala Primary Hospital, Northwestern Ethiopia. BMC Pregnancy Childbirth.

[12] Lu H., Cao W., Zhang L., Yang L., Bi X., Lin Y., Deng W., Jiang T., Sun F., Zeng Z. (2023). Effects of hepatitis B virus infection and strategies for preventing mother-to-child transmission on maternal and fetal T-cell immunity. Front. Immunol..

[13] Tsai K.N., Kuo C.F., Ou J.J. (2018). Mechanisms of Hepatitis B Virus Persistence. Trends Microbiol..

[14] Păcurar D., Dinulescu A., Jugulete G., Păsărică A.-S., Dijmărescu I. (2024). Hepatitis B in Pediatric Population: Observational Retrospective Study in Romania. Life.

[15] Schillie S., Vellozzi C., Reingold A., Harris A., Haber P., Ward J.W., Nelson N.P. (2018). Prevention of Hepatitis B Virus Infection in the United States: Recommendations of the Advisory Committee on Immunization Practices. MMWR Recomm. Rep..

[16] Niţă A.F., Păcurar D. (2019). Adequacy of scoring systems in diagnosing paediatric autoimmune hepatitis: Retrospective study using a control group children with Hepatitis B infection. Acta Paediatr..

[17] (2017). Global Hepatitis Report 2017.

[18] Wu J., Wang H., Xiang Z., Jiang C., Xu Y., Zhai G., Ling Z., Chinese Consortium for the Study of Hepatitis E (CCSHE) (2024). Role of viral hepatitis in pregnancy its triggering mechanism. J. Transl. Int. Med..

[19] Fujiko M., Chalid M.T., Turyadi Ie S.I., Maghfira Syafri Wahyuni R., Roni M., Patellongi I., Massi M.N., Muljono D.H. (2015). Chronic hepatitis B in pregnant women: Is hepatitis B surface antigen quantification useful for viral load prediction?. Int. J. Infect. Dis..

[20] di Filippo Villa D., Navas M.C. (2023). Vertical Transmission of Hepatitis B Virus-An Update. Microorganisms.

[21] Sintusek P., Wanlapakorn N., Poovorawan Y. (2023). Strategies to Prevent Mother-to-child Transmission of Hepatitis B Virus. J. Clin. Transl. Hepatol..

[22] Jaramillo C.M., De La Hoz F., Porras A., Di Filippo D., Choconta-Piraquive L.A., Payares E., Montes N., Navas M.C. (2017). Characterization of Hepatitis B Virus in Amerindian Children and Mothers from Amazonas State, Colombia. PLoS ONE.

[23] Liu J.F., Chen T.Y., Zhao Y.R. (2021). Vertical transmission of hepatitis B virus: Propositions and future directions. Chin. Med. J..

[24] Veronese P., Dodi I., Esposito S., Indolfi G. (2021). Prevention of vertical transmission of hepa titis B virus infection. World J. Gastroenterol..

[25] Tovo P.A., Calitri C., Scolfaro C., Gabiano C., Garazzino S. (2016). Vertically acquired hepatitis C virus infection: Correlates of transmission and disease progression. World J. Gastroenterol..

[26] El-Shabrawi M.H.F., Kamal N.M., Mogahed E.A., Elhusseini M.A., Aljabri M.F. (2019). Perinatal transmission of hepatitis C virus: An update. Arch. Med. Sci..

[27] Le Campion A., Larouche A., Fauteux-Daniel S., Soudeyns H. (2012). Pathogenesis of hepatitis C during pregnancy and childhood. Viruses.

[28] Dustin L.B., Bartolini B., Capobianchi M.R., Pistello M. (2016). Hepatitis C virus: Life cycle in cells, infection and host response, and analysis of molecular markers influencing the outcome of infection and response to therapy. Clin. Microbiol. Infect..

[29] Liu Z., Chen Z., Cui F., Ding Y., Gao Y., Han G., Jia J., Li J., Li Z., Liu Y. (2022). Management Algorithm for Prevention of Mother-to-child Transmission of Hepatitis B Virus (2022). J. Clin. Transl. Hepatol..

[30] Jhaveri R., Hashem M., El-Kamary S.S., Saleh D.A., Sharaf S.A., El-Mougy F., Abdelsalam L., Ehab M., El-Ghazaly H. (2015). Hepatitis C Virus (HCV) Vertical Transmission in 12-Month-Old Infants Born to HCV-Infected Women and Assessment of Maternal Risk Factors. Open Forum Infect. Dis..

[31] Lee L.Y., Lee G.H., Mattar C., Saw S., Aw M. (2019). Maternal HBeAg positivity and viremia associated with umbilical cord blood hepatitis B viremia. Pediatr. Neonatol..

[32] Mavilia M.G., Wu G.Y. (2017). Mechanisms and Prevention of Vertical Transmission in Chronic Viral Hepatitis. J. Clin. Transl. Hepatol..

[33] Li H., Sushmitha S., Kumar R. (2025). Management and Outcomes of Chronic Hepatitis B in Pregnancy: A Retrospective Study from a Tertiary Center in Singapore. Cureus.

[34] Walker T.Y., Smith E.A., Fenlon N., Lazaroff J.E., Dusek C., Fineis P., Crowley S.A., Benson R., Veselsky S.L., Murphy T.V. (2016). Characteristics of Pregnant Women with Hepatitis B Virus Infection in 5 US Public Health Jurisdictions, 2008–2012. Public Health Rep..

[35] Matthews P.C., Ocama P., Wang S., El-Sayed M., Turkova A., Ford D., Torimiro J., Garcia Ferreira A.C., Espinosa Miranda A., De La Hoz Restrepo F.P. (2023). Enhancing interventions for prevention of mother-to-child- transmission of hepatitis B virus. JHEP Rep..

[36] Hu Y., Yu H. (2020). Prevention strategies of mother-to-child transmission of hepatitis B virus (HBV) infection. Pediatr. Investig..

[37] Yi P., Chen R., Huang Y., Zhou R.R., Fan X.G. (2016). Management of mother-to-child transmission of hepatitis B virus: Propositions and challenges. J. Clin. Virol..

[38] Sayre W., Thompson P. (2021). Prevention of Vertical Transmission of Hepatitis B Within a North Carolina Hospital System. Clin. Ther..

[39] Aslam A., Campoverde Reyes K.J., Malladi V.R., Ishtiaq R., Lau D.T.Y. (2018). Management of chronic hepatitis B during pregnancy. Gastroenterol. Rep..

[40] Maraolo A.E., Gentile I., Buonomo A.R., Pinchera B., Borgia G. (2018). Current evidence on the management of hepatitis B in pregnancy. World J. Hepatol..

[41] Pan C.Q., Zhu B.S., Xu J.P., Li J.X., Sun L.J., Tian H.X., Zhang X.H., Li S.W., Dai E.H. (2022). Pregnancy and fetal outcomes of chronic hepatitis C mothers with viremia in China. World J. Gastroenterol..

[42] Post J.J. (2017). Update on hepatitis C and implications for pregnancy. Obstet. Med..

[43] Dieye N.L., Varol M., Zorich S.C., Millen A.E., Yu K.O.A., Gómez-Duarte O.G. (2023). Retrospective analysis of vertical Hepatitis C exposure and infection in children in Western New York. BMC Gastroenterol..

[44] Epstein R.L., Sabharwal V., Wachman E.M., Saia K.A., Vellozzi C., Hariri S., Linas B.P. (2018). Perinatal Transmission of Hepatitis C Virus: Defining the Cascade of Care. J. Pediatr..

[45] Aebi-Popp K., Duppenthaler A., Rauch A., De Gottardi A., Kahlert C. (2016). Vertical transmission of hepatitis C: Towards universal antenatal screening in the era of new direct acting antivirals (DAAs)? Short review and analysis of the situation in Switzerland. J. Virus Erad..

[46] Ades A.E., Gordon F., Scott K., Collins I.J., Claire T., Pembrey L., Chappell E., Mariné-Barjoan E., Butler K., Indolfi G. (2023). Overall Vertical Transmission of Hepatitis C Virus, Transmission Net of Clearance, and Timing of Transmission. Clin. Infect. Dis..

[47] Tziomalos K., Neokosmidis G., Mavromatidis G., Dinas K. (2018). Novel insights in the prevention of perinatal transmission of hepatitis B. World J. Hepatol..

[48] McCluskey J.M., Sato A.I. (2025). Vertical Transplacental Infections. StatPearls [Internet].

[49] Vyas A.K., Negi P., Patra S., Maras J.S., Ramakrishna G., Sarin S.K., Trehanpati N. (2019). Maternal Immunity Influences Vertical Transmission of Hepatitis B to Newborns. Hepatol. Commun..

[50] Liu C.P., Zeng Y.L., Zhou M., Chen L.L., Hu R., Wang L., Tang H. (2015). Factors associated with mother-to-child transmission of hepatitis B virus despite immunoprophylaxis. Intern. Med..

[51] Xu Y.Y., Liu H.H., Zhong Y.W., Liu C., Wang Y., Jia L.L., Qiao F., Li X.X., Zhang C.F., Li S.L. (2015). Peripheral Blood Mononuclear Cell Traffic Plays a Crucial Role in Mother-to-Infant Transmission of Hepatitis B Virus. Int. J. Biol. Sci..

[52] Eke A.C., Eleje G.U., Eke U.A., Xia Y., Liu J. (2017). Hepatitis B immunoglobulin during pregnancy for prevention of mother-to-child transmission of hepatitis B virus. Cochrane Database Syst. Rev..

[53] Hu X., Yang Y., Feng G., Zhou X., Tang M., Yan H., Li M., Liu A., Zhu Y. (2024). Hepatitis B virus in oocytes and embryos: Pregnancy outcomes and children’s health. F S Rep..

[54] Jin L., Nie R., Li Y., Xiao N., Zhu L., Zhu G. (2016). Hepatitis B surface antigen in oocytes and embryos may not result in vertical transmission to offspring of hepatitis B virus carriers. Fertil. Steril..

[55] Benova L., Awad S.F., Miller F.D., Abu-Raddad L.J. (2015). Estimation of hepatitis C virus infections resulting from vertical transmission in Egypt. Hepatology.

[56] Sheng Q., Ding Y., Li B., Han C., Li Y., Zhang C., Bai H., Wang J., Zhao L., Xia T. (2018). Efficacy and safety of nucleos(t)ide analogues to prevent hepatitis B virus mother-to-child transmission in pregnant women with high viremia: Real life practice from China. Int. J. Med. Sci..

[57] Chen X., Chen J., Wen J., Xu C., Zhang S., Zhou Y.H., Hu Y. (2013). Breastfeeding is not a risk factor for mother-to-child transmission of hepatitis B virus. PLoS ONE.

[58] Funk A.L., Lu Y., Yoshida K., Zhao T., Boucheron P., van Holten J., Chou R., Bulterys M., Shimakawa Y. (2021). Efficacy and safety of antiviral prophylaxis during pregnancy to prevent mother-to-child transmission of hepatitis B virus: A systematic review and meta-analysis. Lancet Infect. Dis..

[59] (2020). Prevention of Mother-to-Child Transmission of Hepatitis B Virus: Guidelines on Antiviral Prophylaxis in Pregnancy.

[60] Giugliano S., Petroff M.G., Warren B.D., Jasti S., Linscheid C., Ward A., Kramer A., Dobrinskikh E., Sheiko M.A., Gale M. (2015). Hepatitis C Virus Sensing by Human Trophoblasts Induces Innate Immune Responses and Recruitment of Maternal NK Cells: Potential Implications for Limiting Vertical Transmission. J. Immunol..

[61] Tsai K.N., Ou J.J. (2021). Hepatitis B virus e antigen and viral persistence. Curr. Opin. Virol..

[62] Ansari A., Vincent J.P., Moorhouse L., Shimakawa Y., Nayagam S. (2023). Risk of early horizontal transmission of hepatitis B virus in children of uninfected mothers in sub-Saharan Africa: A systematic review and meta-analysis. Lancet Glob. Health.

[63] Hou J., Cui F., Ding Y., Dou X., Duan Z., Han G., Jia J., Mao Q., Li J., Li Z. (2019). Management Algorithm for Interrupting Mother-to-Child Transmission of Hepatitis B Virus. Clin. Gastroenterol. Hepatol..

[64] Machaira M., Papaevangelou V., Vouloumanou E.K., Tansarli G.S., Falagas M.E. (2015). Hepatitis B Vaccine Alone or with Hepatitis B Immunoglobulin in Neonates of HBsAg+/HBeAg− Mothers: A Systematic Review and Meta-Analysis. J. Antimicrob. Chemother..

[65] Stevens C.E., Toy P., Kamili S., Taylor P.E., Tong M.J., Xia G.L., Vyas G.N. (2017). Eradicating hepatitis B virus: The critical role of preventing perinatal transmission. Biologicals.

[66] Joshi S.S., Gao S., Castillo E., Coffin C.S. (2020). Presence of Precore (C)/C Promoter Mutants in Peripheral Blood Mononuclear Cells of Chronic Hepatitis B (CHB) Carriers During Pregnancy Does Not Correlate with Increased Risk of Liver Disease in 4 Years of Follow-Up. Dig. Dis. Sci..

[67] Yin Y., Zhang P., Tan Z., Zhou J., Wu L., Hou H. (2016). The Association of Pre-S/S Gene Mutations and Hepatitis B Virus Vertical Transmission. Hepat. Mon..

[68] He R., Wen P., Xiong M., Fan Z., Li F., Luo D., Xie X. (2022). Cesarean section in reducing mother-to-child HBV transmission: A meta-analysis. J. Matern. Fetal Neonatal Med..

[69] Chen H.L., Cai J.Y., Song Y.P., Zha M.L., Qin G. (2019). Vaginal delivery and HBV mother to child transmission risk after immunoprophylaxis: A systematic review and a meta-analysis. Midwifery.

[70] Yang M., Qin Q., Fang Q., Jiang L., Nie S. (2017). Cesarean section to prevent mother-to-child transmission of hepatitis B virus in China: A meta-analysis. BMC Pregnancy Childbirth.

[71] Chasela C.S., Kourtis A.P., Wall P., Drobeniuc J., King C.C., Thai H., Teshale E.H., Hosseinipour M., Ellington S., Codd M.B. (2014). Hepatitis B virus infection among HIV-infected pregnant women in Malawi and transmission to infants. J. Hepatol..

[72] Sookoian S. (2006). Liver disease during pregnancy: Acute viral hepatitis. Ann. Hepatol..

[73] Stasi C., Milli C., Voller F., Silvestri C. (2024). The Epidemiology of Chronic Hepatitis C: Where We Are Now. Livers.

[74] Inui A., Fujisawa T., Sogo T., Komatsu H., Isozaki A., Sekine I. (2002). Different outcomes of vertical transmission of hepatitis C virus in a twin pregnancy. J. Gastroenterol. Hepatol..

[75] Thompson P., Morgan C.E., Ngimbi P., Mwandagalirwa K., Ravelomanana N.L.R., Tabala M., Fathy M., Kawende B., Muwonga J., Misingi P. (2021). Arresting vertical transmission of hepatitis B virus (AVERT-HBV) in pregnant women and their neonates in the Democratic Republic of the Congo: A feasibility study. Lancet Glob. Health..

[76] World Health Organization (WHO) Hepatitis B. https://www.who.int/news-room/fact-sheets/detail/hepatitis-b.

[77] (2017). Guidelines on Hepatitis B and C Testing.

[78] Perinatal Hepatitis B Prevention Program Manual. https://doh.wa.gov/sites/default/files/legacy/Documents/Pubs//348-165_PerinatalHepatitisBPreventionProgramGuidelines.pdf#:~:text=Post%2DVaccination%20Serology%20Testing%20PVST%20helps%20identify%20infants,vaccine%20series%20if%20the%20series%20is%20delayed).

[79] Management of Infants Born to Women with Hepatitis B Virus Infection for Pediatricians. https://www.cdc.gov/vaccines/programs/perinatal-hepb/downloads/HepB-Provider-tipsheet-508.pdf.

[80] Tamandjou Tchuem C.R., Andersson M.I., Wiysonge C.S., Mufenda J., Preiser W., Cleary S. (2021). Prevention of hepatitis B mother-to-child transmission in Namibia: A cost- effectiveness analysis. Vaccine.

[81] Dionne-Odom J., Njei B., Tita A.T.N. (2018). Elimination of Vertical Transmission of Hepatitis B in Africa: A Review of Available Tools and New Opportunities. Clin. Ther..

[82] Breakwell L., Tevi-Benissan C., Childs L., Mihigo R., Tohme R. (2017). The Status of Hepatitis B Control in the African Region. Pan Afr. Med. J..

[83] Nayagam S., de Villiers M.J., Shimakawa Y., Lemoine M., Thursz M.R., Walsh N., Hallett T.B. (2023). Impact and cost-effectiveness of hepatitis B virus prophylaxis in pregnancy: A dynamic simulation modelling study. Lancet Gastroenterol. Hepatol..

[84] Enebe J.T., Enebe N.O., Onwujekwe O.E. (2025). Willingness to pay for hepatitis B immunoglobulin among pregnant women in Enugu metropolis, South-East, Nigeria: A cross-sectional study. BMC Pregnancy Childbirth.

[85] Dionne-Odom J., Cozzi G.D., Franco R.A., Njei B., Tita A.T.N. (2022). Treatment and prevention of viral hepatitis in pregnancy. Am. J. Obstet. Gynecol..

[86] Bierhoff M., Nelson K.E., Guo N., Jia Y., Angkurawaranon C., Jittamala P., Carrara V., Watthanaworawit W., Ling C., Tongprasert F. (2020). Prevention of mother-to-child transmission of hepatitis B virus: Protocol for a one-arm, open-label intervention study to estimate the optimal timing of tenofovir in pregnancy. BMJ Open.

[87] Nguyen H.T., Thavorncharoensap M., Phung T.L., Anothaisintawee T., Chaikledkaew U., Sobhonslidsuk A., Talungchit P., Chaiyakunapruk N., Attia J., McKay G.J. (2022). Comparative efficacy and safety of pharmacologic interventions to prevent mother-to-child transmission of hepatitis B virus: A systematic review and network meta-analysis. Am. J. Obstet. Gynecol..

[88] Pan X., Zhou L., Hu J., Zhai P., Ou X., He F., Pan C.Q. (2025). Tenofovir Alafenamide Therapy Throughout Pregnancy in Mothers with Hepatitis B. Aliment. Pharmacol. Ther..

[89] Pan C.Q. (2022). The role of tenofovir disoproxil fumarate for preventing vertical transmission of hepatitis B. Antivir. Ther..

[90] Delamare H., Ishii-Rousseau J.E., Rao A., Cresta M., Vincent J.P., Ségéral O., Nayagam S., Shimakawa Y. (2024). Proportion of pregnant women with HBV infection eligible for antiviral prophylaxis to prevent vertical transmission: A systematic review and meta-analysis. JHEP Rep..

[91] Garcia D., Porras A., Rico Mendoza A., Alvis N., Navas M.C., De La Hoz F., De Neira M., Osorio E., Valderrama J.F. (2018). Hepatitis B Infection Control in Colombian Amazon after 15 Years of Hepatitis B Vaccination. Effectiveness of Birth Dose and Current Prevalence. Vaccine.

[92] Guidance on the Hepatitis B Antenatal Screening and Selective Neonatal Immunization Pathway. Updated 13 June 2025. https://www.gov.uk/government/publications/hepatitis-b-antenatal-screening-and-selective-neonatal-immunisation-pathway/guidance-on-the-hepatitis-b-antenatal-screening-and-selective-neonatal-immunisation-pathway--2#:~:text=The%20DBS%20sample%20will%20be%20tested%20for,test%20for%20antibody%20response%20to%20vaccination%20(anti%2DHBs).

[93] Meng X., Zhang W., Yan B., Feng Y., Lu J., Zhang L. (2025). Progress toward elimination mother-to-child transmission of HBV in Shandong Province, China: Coverage trend in hepatitis B vaccination and HBIG administration, 2017–2024. Hum. Vaccines Immunother..

[94] ECDC Surveillance Report Hepatitis B Annual Epidemiological Report for 2022. https://www.ecdc.europa.eu/sites/default/files/documents/AER%20HEPB%202022_0.pdf.

[95] Panagiotakopoulos L., Sandul A.L., Conners E.E., Foster M.A., Nelson N.P., Wester C. (2023). CDC Recommendations for Hepatitis C Testing Among Perinatally Exposed Infants and Children—United States, 2023. Recomm. Rep..

[96] Thilakanathan C., Wark G., Maley M., Davison S., Lawler J., Lee A., Shackel N., Nguyen V., Jackson K., Glass A. (2018). Mother to Child Transmission of Hepatitis B: Examining Viral Cut Offs, Maternal HBsAg Serology and Infant Testing. Liver Int..

[97] Biondi M.J., Flemming J., van Gennip J., Barnett T., Masterman C., Mendlowitz A., Mooney M., Fontaine G., Feld J.J. (2025). Hepatitis C virus testing in infants, a move to early screening by HCV RNA at 2 months of age. Paediatr. Child. Health.

[98] Hachicha-Maalej N., Lepers C., Collins I.J., Mostafa A., Ades A.E., Judd A., Scott K., Gibb D.M., Pett S., Indolfi G. (2024). Modelling the potential clinical and economic impact of universal antenatal hepatitis C (HCV) screening and providing treatment for pregnant women with HCV and their infants in Egypt: A cost-effectiveness study. BMJ Public Health.

[99] Liu Z., Li M., Hutton D.W., Wagner A.L., Yao Y., Zhu W., Cao L., Tang S., Pan J., Wang Y. (2022). Impact of the national hepatitis B immunization program in China: A modeling study. Infect. Dis. Poverty.

[100] Liu J., Liang W., Jing W., Liu M. (2019). Countdown to 2030: Eliminating hepatitis B disease, China. Bull. World Health Organ..

[101] Yan B., Zhang X., Lv J., Feng Y., Meng X., Lin X., Zhang Y., Wang S., Ji F., Chen M. (2024). Seroprevalence of hepatitis B among the general population in Shandong Province, Eastern China, an update 30 years after the implementation of the neonatal vaccination program. BMC Infect. Dis..

[102] Ni Y.H., Chang M.H., Jan C.F., Hsu H.Y., Chen H.L., Wu J.F., Chen D.S. (2016). Continuing Decrease in Hepatitis B Virus Infection 30 Years After Initiation of Infant Vaccination Program in Taiwan. Clin. Gastroenterol. Hepatol..

