# Supplementary material for: Vertical Transmission of Hepatitis B and C—Then and Now—A Comprehensive Literature Systematic Review
Source: Viruses. 2025 Oct 20;17(10):1395. doi: 10.3390/v17101395 (PMC12567994; doi:10.3390/v17101395)
Supplement: Supplementary file 1 [file viruses-17-01395-s001.zip › viruses-3921951-supplementary.pdf]

PRISMA 2020 flow diagram for new systematic reviews which included searches of databases, registers and other sources

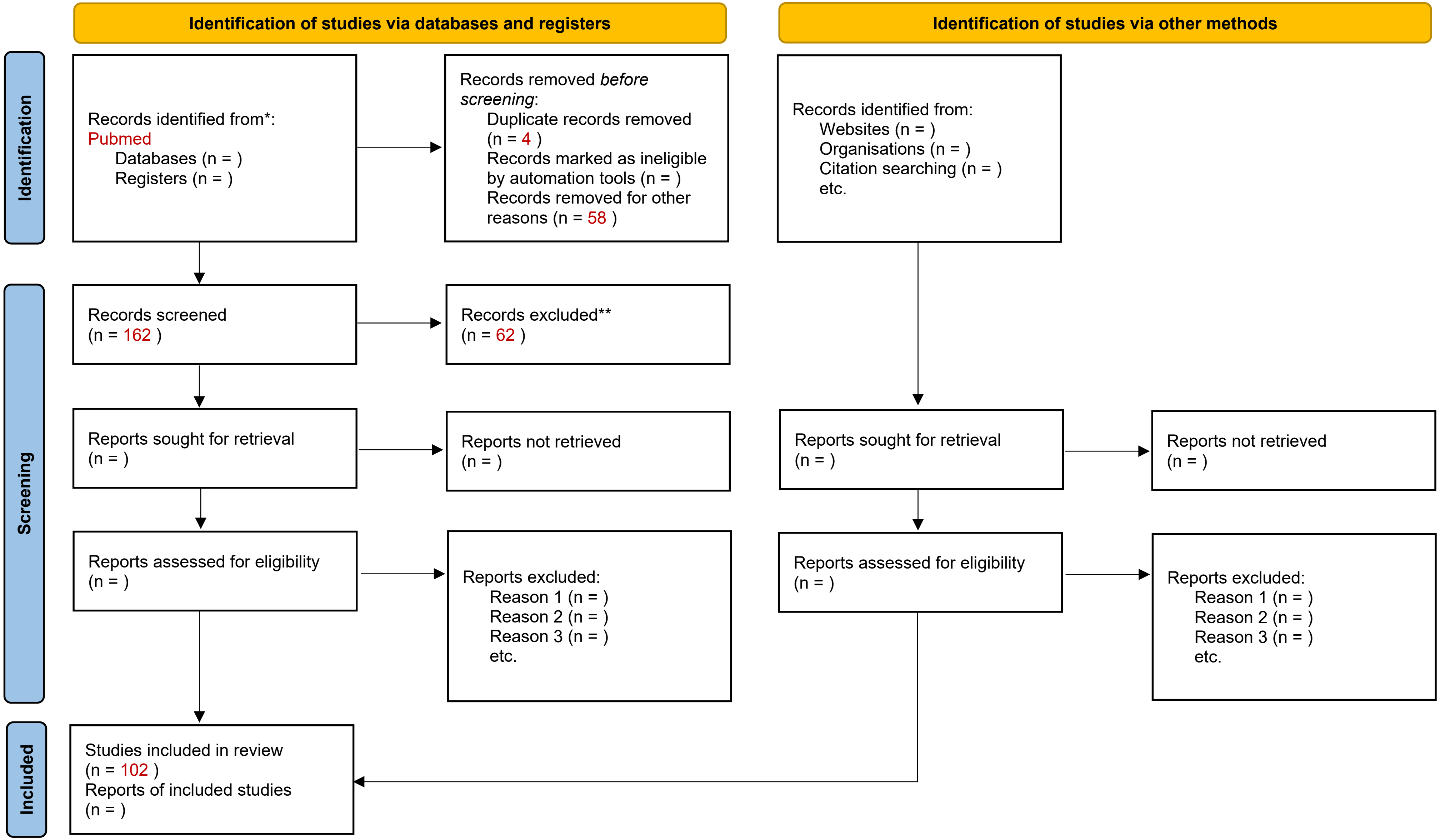

*Consider, if feasible to do so, reporting the number of records identified from each database or register searched (rather than the total number across all databases/registers).
**If automation tools were used, indicate how many records were excluded by a human and how many were excluded by automation tools.

This work is licensed under CC BY 4.0. To view a copy of this license, visit https://creativecommons.org/licenses/by/4.0/
